# LncRNA Dleu2 Serve as a Novel Biomarker for Ablation Recurrence and Promote Atrial Remodelling by Targeting Nr4a1 in Atrial Fibrillation

**DOI:** 10.1111/jcmm.70618

**Published:** 2025-06-12

**Authors:** Feiyu Wei, Yazhe Ma, Hong Xiang, Xi Zhang, Jie Fan

**Affiliations:** ^1^ Department of Cardiology The First People's Hospital of Yunnan Province, The Affiliated Hospital of Kunming University of Science and Technology Kunming Yunnan People's Republic of China

**Keywords:** atrial fibrillation, atrial fibrosis, atrial remodelling, LncRNA Dleu2, recurrence

## Abstract

Atrial remodelling is the principal pathological mechanism for atrial fibrillation (AF) development and progression. Long noncoding RNAs (LncRNAs) exhibit important effects on cardiovascular diseases. However, the role of LncRNAs in AF development requires further investigation. This study aimed to explore the function and mechanism of LncRNAs in AF. The differentially expressed LncRNAs of atrial tissue in a mouse AF model, which was established via continuous infusion of Ang II for 3 weeks, were screened with RNA sequencing. Experiments included an electrophysiological study; Masson, H&E and TUNEL staining; flow cytometry; and RNA pull‐down; FISH and RNA immunoprecipitation assays were performed to define the function and underlying mechanisms of LncRNAs in AF susceptibility and atrial remodelling. The Kaplan–Meier method was used to plot the curve of freedom from atrial tachyarrhythmia. LncRNA Dleu2 expression was increased in atrial tissue and peripheral blood and was positively associated with left atrial fibrosis in persistent AF. Furthermore, elevated expression of LncRNA Dleu2 was correlated with a higher AF recurrence rate after ablation at the 24‐month follow‐up (65.0% vs. 85.0%, *p* = 0.03). Accordingly, upregulation and downregulation of LncRNA Dleu2 expression could regulate atrial remodelling and AF susceptibility, and we also demonstrated that LncRNA Dleu2 directly bound to Nr4a1. Subsequently, inhibition of Nr4a1 expression could also regulate AF susceptibility and atrial remodelling and reverse the effects of LncRNA Dleu2 on AF occurrence. This study demonstrated that LncRNA Dleu2 was independently associated with atrial fibrosis and AF recurrence after ablation, and contributed to AF susceptibility by directly targeting Nr4a1.

## 1 Introduction

Atrial fibrillation (AF) is a prevalent rapid atrial arrhythmia, which increases the risk of heart failure and systemic embolism [1]. Successful catheter ablation has achieved satisfactory outcomes in paroxysmal AF [2], but the recurrence rate continues to be high in non‐paroxysmal AF [3]. The extent of left atrial fibrosis in non‐paroxysmal AF is significantly more extensive than in paroxysmal AF and is positively correlated with AF recurrence after radiofrequency catheter ablation (RFCA) [4]. Furthermore, sinus rhythm maintenance was increased with pulmonary vein isolation (PVI) plus substrate modification based on low‐voltage area caused by atrial fibrosis compared with PVI [5].

Atrial electrical remodelling, characterised by ectopy triggers and abnormal automaticity, is the main role during AF onset. Then atrial structural remodelling, characterised by cardiomyocyte apoptosis and abnormal fibroblast proliferation, is the main pathological process during AF progression [6]. Atrial cardiomyocyte apoptosis leads to abnormal cellular electrical activity and myocardial fibrosis [7]. Increasing evidence has shown that cardiac and systemic inflammatory responses are the independent risk factors for AF onset and progression [8]. An inflammatory response can promote atrial remodelling by mediating fibroblast proliferation, tissue fibrosis, cellular oxidative stress and cardiomyocyte apoptosis [9]. Tumour necrosis factor‐α (TNF‐α) is an important inflammatory factor and its enhanced expression induces an acute or chronic inflammation response. TNF‐α expression is enhanced in AF and is closely associated with the AF type, course and prognosis [10, 11]. High cardiac expression of TNF‐α results in abnormal calcium processing in atrial cardiomyocytes and decreases atrial contractility, increasing AF susceptibility [12]. Similarly, studies have also demonstrated that IL‐6 and IL‐12 are associated with AF and contribute to atrial remodelling [13, 14].

The Long noncoding RNA (LncRNA) is a noncoding RNA molecule between 200 and 100,000 nucleotides in length [15]. Studies demonstrated LncRNAs are correlated with AF onset and progression. For example, LncRNA PVT1 contributes to atrial structural remodelling and AF development via SP1 to activate the TGF‐β/Smad3 signalling pathway [16]. TCONS_00075467 regulates atrial electrical remodelling by affecting calcium channel protein CACNA1C [17]. In addition, LncRNAs are involved in regulating TNF‐α expression, cardiomyocyte death and other pathological processes [18]. However, LncRNAs, which are related to atrial fibrosis and inflammatory response, have been rarely reported in AF. Similarly, there are few reports describing how inflammatory response‐related LncRNAs regulate AF development.

Therefore, the present study first aimed to screen the differentially expressed LncRNA in patients with AF compared with control subjects by RNA sequencing. Subsequently, bioinformatics analysis was conducted on these differentially expressed LncRNAs to identify those associated with atrial fibrosis and inflammatory responses, and also analysed the correlation between these differences in expression of LncRNAs and AF recurrence after RFCA. We found LncRNA Dleu2 expression was upregulated in AF compared with control, and LncRNA Dleu2 expression was positively associated with left atrial fibrosis and inflammatory response factor, and also was associated with AF recurrence after catheter ablation. Finally, we investigated the potential function and mechanism of action of LncRNA Dleu2 on atrial remodelling and AF development in an in vivo and in vitro model; the results showed aberrant expression of LncRNA Dleu2 contributed to atrial remodelling and AF development through regulating myocardial cell apoptosis and inflammatory response by targeting Nr4a1. Our present study demonstrated that LncRNA Dleu2 may become a biomarker for atrial fibrosis and recurrence prediction after AF ablation.

## 2 Methods and Materials

### 2.1 Animals and Treatments

Male C57BL/6 mice (aged 6–8 weeks, 20–25 g) were purchased from SLAC (Shanghai, China). The mouse AF model was established by injecting acetylcholine (ACh)‐CaCl2 for 7 days according to our previously described methods [19], or via continuous infusing Ang II (2000 ng/kg/min) for three weeks using an osmotic micropump [20] (experimental details are presented in Supporting Information S1), while the control group was injected with 0.9% PBS. An adenovirus vector carrying the targeting LncRNA (Sh‐Dleu2 and ADV‐Dleu2) and Nr4a1 (Sh‐Nr4a1 and ADV‐Nr4a1) was injected to over‐express or down‐express the expression of LncRNA Dleu2 and Nr4a1 via tail‐vein injection, with repeated injections every 7 days. Animal experimentation was conducted in accordance with the Guide for the Care and Use of Laboratory Animals, and all study procedures were approved by the Institutional Animal Care and Use Committee of Kunming University of Science and Technology (HB1911020).

### 2.2 RNA Sequencing

The RNA was isolated from atrial tissue of AF model mice and the control group. A chain‐specific library was then constructed by removing ribosomal RNA (rRNA depletion). After passing quality inspection of the library, we employed an Illumina NovaSeq 6000 for RNA sequencing, with a sequencing read length of 2 × 150 bp (PE150) at both ends (the detailed sequencing procedure is presented in Supporting Information S1).

### 2.3 Study Populations

We consecutively included 80 patients with AF who were hospitalised in the Cardiology Department of The First People's Hospital of Yunnan Province from October 2018 to December 2019. Then 80 baseline‐matched individuals without evidence of AF were included as controls during the same period. Peripheral blood was subsequently collected to detect plasma LncRNA expression level. Nine AF and nine non‐AF patients who underwent cardiac surgical operation were also included. The atrial tissue was collected to detect tissue LncRNA expression level. The detailed inclusion and exclusion criteria are described in the Supplemental Methods. All human studies were approved by the Ethics Committees of the First People's Hospital of Yunnan Province (KHLL2018‐KY089) and conformed to the ethical standards promulgated by the 1964 Declaration of Helsinki and its later amendments.

### 2.4 Isolation of Cardiomyocytes and Treatments

The atrial cardiomyocytes were separated from 1 to 3‐day‐old neonatal mice by differential centrifugation with Percoll delamination solution (Sigma, USA). Mouse heart was minced and digested in PBS that contained 0.1% trypsin (Gibco, BRL) and 0.05% type I collagenase (Gibco, BRL). The digested solution was centrifuged for 5 min and suspended in DMEM (Gibco, BRL) containing 15% fetal bovine serum (Gibco, BRL). The cardiomyocytes were purified with Percoll fluid (Sigma, USA) according to manufacturer instructions. The isolated cardiomyocytes were cultured with DMEM at 37°C and 5% CO_2_. Cardiomyocyte apoptosis was induced with Ang II [21]; the control group was treated with 0.9% PBS. The cardiomyocyte apoptotic rate was detected with flow cytometry (details are presented in Supporting Information S1).

### 2.5 Histological Staining

We conducted Masson and H&E staining to evaluate tissue morphology and atrial fibrosis (procedures were described as our previous study [19]). TUNEL staining was performed to detect myocardial cell apoptosis. Atrial tissue was isolated and imbedded in paraffin and fixed with 4% paraformaldehyde; subsequently, Masson, H&E and TUNEL staining was performed according to the manufacturer's instructions. Sections were ultimately taken using an optical microscope (BX43, Olympus, Tokyo, Japan).

### 2.6 ELISA

The concentrations of TNF‐α, IL‐12 and IL‐6 in plasma and tissues were measured using a commercial ELISA kit (Jianglaibio, JL11794, Shanghai, China) according to the manufacturer's instructions. Briefly, the ELISA kit and prepared samples were equilibrated to room temperature (25°C–28°C) prior to use. Standards or samples (100 μL per well) were added to the wells, and the plate was covered with sealing film and incubated at 25°C–28°C for 120 min. Each well was then washed with 300 μL of washing buffer (5 × 30 s). After the final wash, the plate was inverted and blotted dry on absorbent paper. Next, 50 μL of biotinylated antibody working solution was added to each well, covered and incubated at 25°C–28°C for 60 min. The washing step was repeated as described above. Subsequently, 50 μL of HRP‐conjugated streptavidin was added to each well, covered and incubated in the dark at 25°C–28°C for 20 min. The plate was washed again five times. TMB substrate (90 μL per well) was added, and the plate was incubated in the dark at 25°C–28°C for 20 min. The reaction was stopped by adding 50 μL of stop solution per well, and the contents were mixed thoroughly. The OD450 value was immediately measured for data analysis using a microplate reader (Plus384, SpectraMax, MD, USA).

### 2.7 Screening and Verifying the LncRNA‐Targeting Protein

An RNA pull‐down was used to obtain the LncRNA Dleu2 direct‐binding protein, and target protein molecular information was analysed by protein mass spectrometry. Protein information was then confirmed by western blotting. Furthermore, RNA immunoprecipitation (RIP) evaluation was used to reversely verify the interaction between the targeting protein and LncRNA Dleu2. Finally, fluorescence in situ hybridization (FISH) and immunofluorescence were performed to determine the subcellular localization of LncRNA Dleu2 and Nr4a1 in cardiomyocytes. The detailed methods for RNA pull‐down, RIP and FISH are described in Supporting Information S1. The detailed LncRNA target gene‐screening strategy is presented in Supporting Information S1.

### 2.8 Left Atrial Fibrosis Evaluation and Linear Ablation Strategy

The left atrial low‐voltage area, which indicated the atrial fibrosis, was mapped with a high‐density mapping catheter (Pentaray Catheter, Biosense Webster; Diamond Bar, CA) under a three‐dimensional mapping system (Carto3 system, Biosense Webster; Diamond Bar, CA) in persistent AF during RFCA as in our previously published study [19]. All the patients were subjected to circumferential PVI. If the patient was still in atrial fibrillation after CPVI, cardioversion was used to convert to sinus rhythm. If the first cardioversion failed to restore sinus rhythm, a second cardioversion was performed. If sinus rhythm was still not achieved after three cardioversion attempts, the patient was excluded from the study. In the present study, 35 out of 40 persistent AF patients successfully restored sinus rhythm after the first cardioversion, while the remaining five patients achieved sinus rhythm following the second cardioversion. Left atrial voltage matrix mapping was performed under sinus rhythm. The local electrogram mapping voltage amplitudes were set at 0.1–0.4 mV in bipolar under sinus rhythm, and < 0.1 mV was defined as the low‐voltage area. The low‐voltage area proportion was counted as a percentage of the low‐voltage area to the left atrial surface area.

In the present study, the criteria for superior vena cava (SVC) isolation included an SVC sleeve length greater than 30 mm or clear evidence of SVC‐originated or triggers AF [22]. The left atrial (LA) anterior wall was ablated in cases with low‐voltage areas in the anterior region. Mitral isthmus ablation was performed for mitral isthmus‐dependent atypical left atrial flutter. Cavo‐tricuspid isthmus ablation was conducted in persistent AF cases unless a definite AF triggering focus was identified. In all persistent AF, LA roof and posterior wall ablation were performed unless sinus rhythm was restored prior to the procedure. For paroxysmal AF, if AF episodes persisted after PVI, the same ablation strategy as for persistent AF was applied.

### 2.9 Follow‐Up

Twenty‐four‐hour Holter electrocardiography was performed in all patients with AF after RFCA at 3, 6, 9,12,18 and 24 months to detect the recurrence of atrial tachyarrhythmia (ATs), and ATs recurrent is defined as AF, atrial tachycardia and other high‐frequency atrial events duration longer than 30 s. Then peripheral blood was collected and RNA was extracted to detect the LncRNA Dleu2 expression.

### 2.10 Quantification and Statistical Analysis

The quantitative data are presented as mean ± SD. Categorical‐distribution variables are shown as absolute or relative frequencies. Student's *t*‐test or one‐way ANOVA followed by Tukey's test was used to compare the differences of the data. The correlation between two quantitative data points were made by applying Pearson's correlation coefficients. The correlation between risk factor and AF occurrence was analysed with logistic regression analysis. We compared the recurrence difference of AF using Kaplan–Meier curves between two groups and analysed times to event endpoints using log‐rank tests. The recurrence risk analysis was tested with the COX regression model. The sensitivity and specificity of the recurrence prediction after AF RFCA were determined with time‐dependent receiver operating characteristic (ROC) curves, and the area under the ROC curve (AUC) was calculated. A *p*‐value < 0.05 was considered to be statistically significant. We implemented SPSS 17.0 (IBM, Armonk, NY, USA) and GraphPad Prism7.0 (San Diego, CA, USA) software for all statistical analyses and graphical presentations, respectively.

## 3 Results

### 3.1 LncRNA Dleu2 Is Positively Associated With AF Occurrence

**FIGURE 1:**
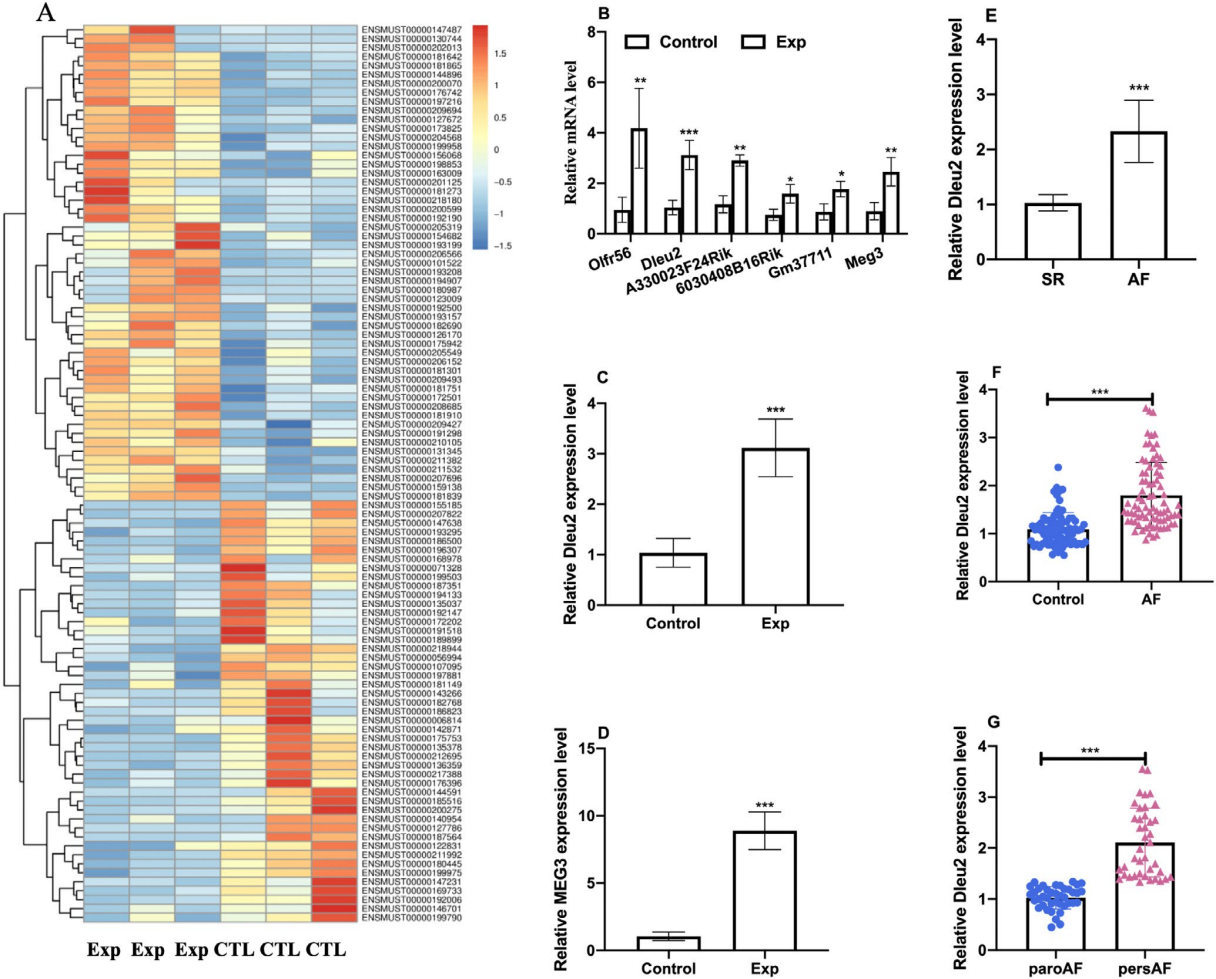
Association between LncRNA Dleu2 and AF occurrence. (A) Differentially expressed LncRNA heatmap in the atrial tissue of mouse AF animal model by transcriptomic sequencing. Exp represents the AF model group and CTL represents the control group. Different colours designate differing amounts of expression (*n* = 3). (B) The expression level of LncRNA Olfr56, LncRNA Dleu2, A330023F24Rik, 6030408B16Rik, Gm37711 and LncRNA MEG3 in the mouse AF animal model (Exp) and Control group (CTL) using qRT‐PCR (*n* = 3). (C) The LncRNA Dleu2 expression in atrial tissue of the Ang II‐induced mice AF animal model (*n* = 6). (D) The atrial tissue LncRNA MEG3 expression level in Ang II‐induced mice AF model (*n* = 6). (E) The atrial tissue LncRNA Dleu2 expression level in AF patients (*n* = 9). (F) Circulating LncRNA Dleu2 levels in AF (*n* = 80). (G) Circulating LncRNA Dleu2 level in paroxysmal AF and persistent AF (*n* = 40). *p < 0.05, **p < 0.01, ***p < 0.001. AF, atrial fibrillation; paro AF, paroxysmal AF; pers. AF, persistent AF; SR, sinus rhythm.

Differentially expressed LncRNAs of the AF mouse model, which was constructed with 7‐day Ach‐CaCl2 induction, are shown with heatmaps in Figure 1A by RNA sequencing. According to the screening criteria, the expression levels of A330023F24Rik, 6030408B16Rik, LncRNA Olfr56, Gm37711, LncRNA Dleu2 and LncRNA MEG3 were increased in the AF model than in the control group. qRT‐PCR results showed the above six distinct LncRNAs were indeed increased in the AF model, which was consistent with the sequencing results (Figure 1B). So as to better validate the role and clinical translation of AF in the future, we focused on the LncRNAs expressed in both mice and humans, and results revealed that LncRNA Dleu2 and LncRNA MEG3 were expressed in both species. Since the AF occurrences were associated with multiple clinical diseases and pathological factors, we also constructed an AF animal model using continuous injection of angiotensin II (Ang II), and observed that LncRNA Dleu2 and LncRNA MEG3 expression levels also significantly increased in the Ang II‐induced AF model (Figure 1C,D). Considering the literature has reported that LncRNA MEG3 was associated with cardiac fibrosis, we explored the function in another study. Hence, we selected the LncRNA Dleu2 as the target molecular for further functional study.

The atrial tissue and plasma expression level of LncRNA Dleu2 in patients with AF and sinus rhythm were also detected, and results demonstrated LncRNA Dleu2 expression was elevated in the atrial tissue as well as in plasma in AF patients (Figure 1E,F). Circulating LncRNA Dleu2 was also augmented in persistent AF compared with paroxysmal AF (Figure 1G). The baseline characteristics of the 80 AF patients and 80 patients without AF evidence were depicted in Tables S1 and S2.

Finally, the results showed the circulating LncRNA Dleu2 level was positively associated with AF development risk by univariate logistic regression, and with the correlation remaining consistent when adjusting for Type2 diabetes mellitus and left atrial diameter in the multivariate‐adjusted model (Table S3).

### 3.2 LncRNA Dleu2 Is Correlated With Left Atrial Fibrosis and Inflammatory Response

**FIGURE 2:**
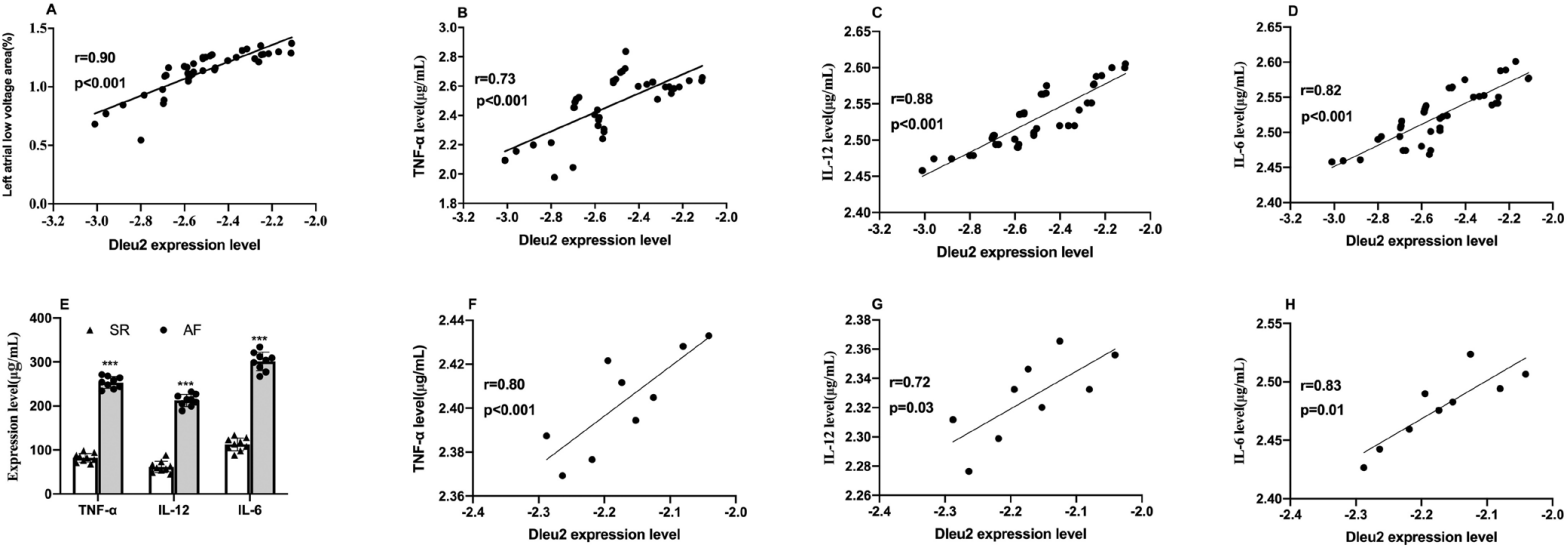
Association between LncRNA Dleu2 expression level and left atrial fibrosis and inflammatory factor. (A) Correlation between LncRNA Dleu2 expression level and left atrial low‐voltage area in persistent AF (*n* = 40). (B–D) The association between LncRNA Dleu2 expression level and TNF‐α, IL‐12, and IL‐6 (n*n* = 40). (E) The expression level of TNF‐α, IL‐12, and IL‐6 in the atrial tissue of patients with AF and SR (*n* = 9). (F–H) The correlation between LncRNA Dleu2 expression and TNF‐α, IL‐12, and IL‐6 level in atrial tissue (*n* = 9). ***p < 0.001. SR, sinus rhythm; AF, atrial fibrillation. The X‐axis units of A–D was 2^−△CT^ and transformed with log10 in accordance with normal distribution.

Pearson's correlation revealed a positive association between the LncRNA Dleu2 level and left atrial fibrosis (Figure 2A). We observed that circulating inflammatory factor levels, which included those of IL‐12, IL‐6 and TNF‐α, were increased with AF compared to controls using ELISA (Table S1). The Pearson correlation results also revealed a positive correlation between the LncRNA Dleu2 level and IL‐12, IL‐6 and TNF‐α levels (Figure 2B–D). Furthermore, the expression levels of IL‐12, IL‐6 and TNF‐α in atrial tissue were also elevated in AF patients compared with individuals showing normal sinus rhythm (Figure 2E), and were positively associated with LncRNA Dleu2 levels in AF (Figure 2F–H).

### 3.3 LncRNA Dleu2 Serves as a Novel Molecular Model to Predict AF Recurrence After Ablation

**FIGURE 3:**
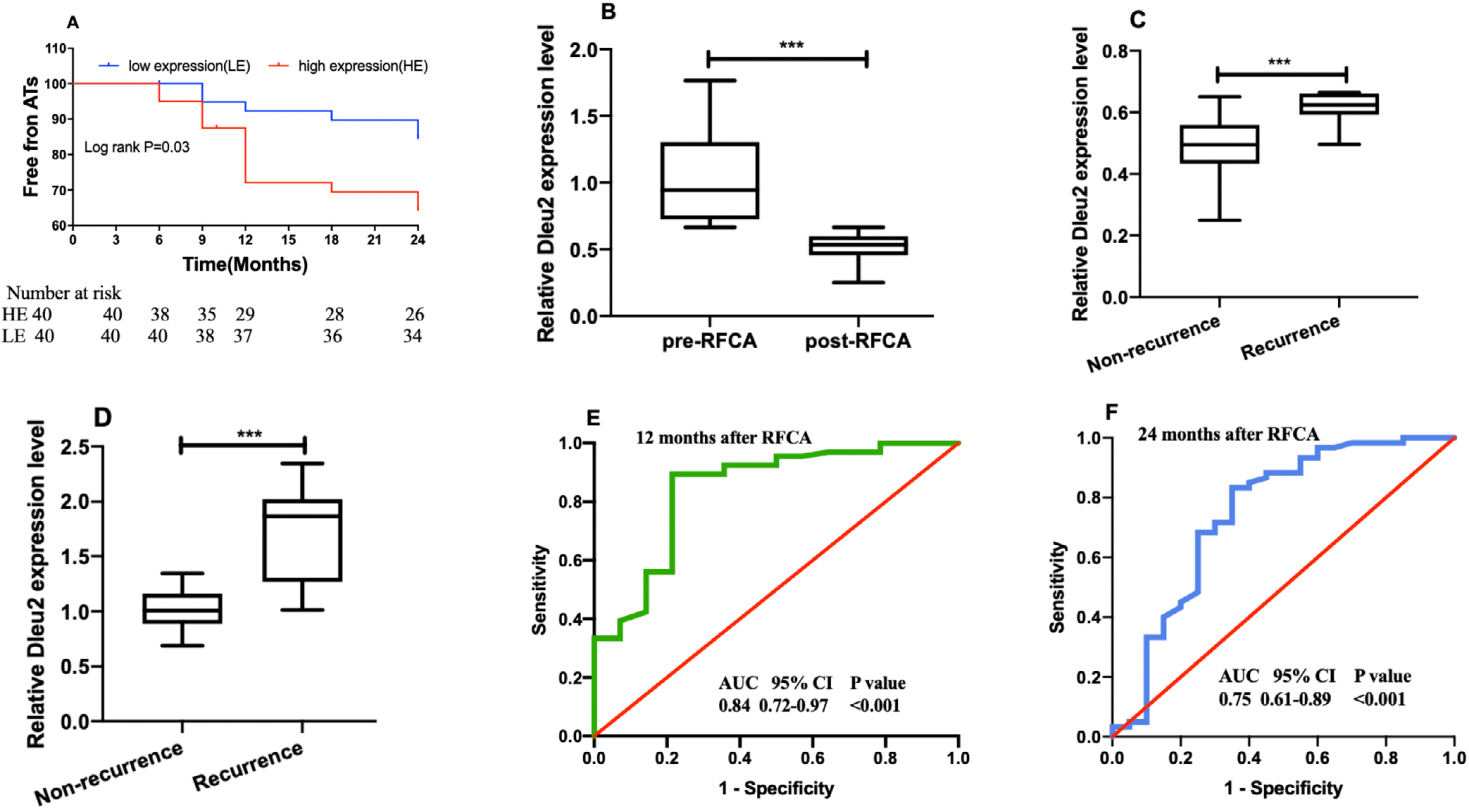
Correlation between LncRNA Dleu2 expression and ATs recurrence after RFCA in AF. (A) Kaplan–Meier curve showing difference in ATs recurrence at different timepoints after RFCA (*n* = 40). (B) Expression change of LncRNA Dleu2 in pre‐RFCA and post‐RFCA (*n* = 40). (C) Expression difference of LncRNA Dleu2 in the recurrence group (*n* = 10) and non‐recurrence group (*n* = 30). (D) Baseline LncRNA Dleu2 expression difference in the AF (recurrence group, *n* = 10) and sinus rhythm maintenance group (*n* = 30). (E) Recurrence predictive ability of the LncRNA Dleu2 using ROC analysis at the 12 months follow‐up (*n* = 40). (F) Recurrence predictive ability of LncRNA Dleu2 using ROC analysis at the 24‐month follow‐up (*n* = 40). ***p < 0.001.

We divided the AF into two groups according to the median expression level of LncRNA Dleu2 in peripheral blood. At 24 months follow‐up, recurrent atrial arrhythmia (atrial tachycardia, AT or AF) occurred in 14 of 40 patients (35.0%) in the high‐expression group and six of 40 (15.0%) in the low‐expression group (a time‐to‐event analysis is presented in Figure 3A). The AT/AF recurrence occurred more often in the LncRNA Dleu2 high expression group than in the low expression group (HR, 2.64; 95% CI, 1.07–9.12; *p* = 0.03). COX regression analysis revealed that high expression of LncRNA Dleu2 was an independent prediction factor for AF recurrence after RFCA (HR, 3.12; 95% CI, 1.24–4.15; *p* = 0.03). Furthermore, RFCA could restore the circulating LncRNA Dleu2 expression level in persistent AF (Figure 3B), and LncRNA Dleu2 expression was maintained at a higher level in the AF recurrence group than in the non‐recurrence group (Figure 3C); the baseline LncRNA expression level was also higher in the AF recurrence group than in the non‐recurrence group (Figure 3D). The predictive ability of LncRNA Dleu2 was 0.84 (AUC, 0.84; 95% CI, 0.72–0.97; *p* < 0.001) at 12 months follow‐up (Figure 3E) and 0.75 (AUC, 0.75; 95% CI, 0.61–0.89; *p* < 0.001) at 24 months follow‐up (Figure 3F), respectively.

### 3.4 Downregulation of LncRNA Dleu2 Expression Inhibits Atrial Remodelling and Reduces AF Susceptibility

**FIGURE 4:**
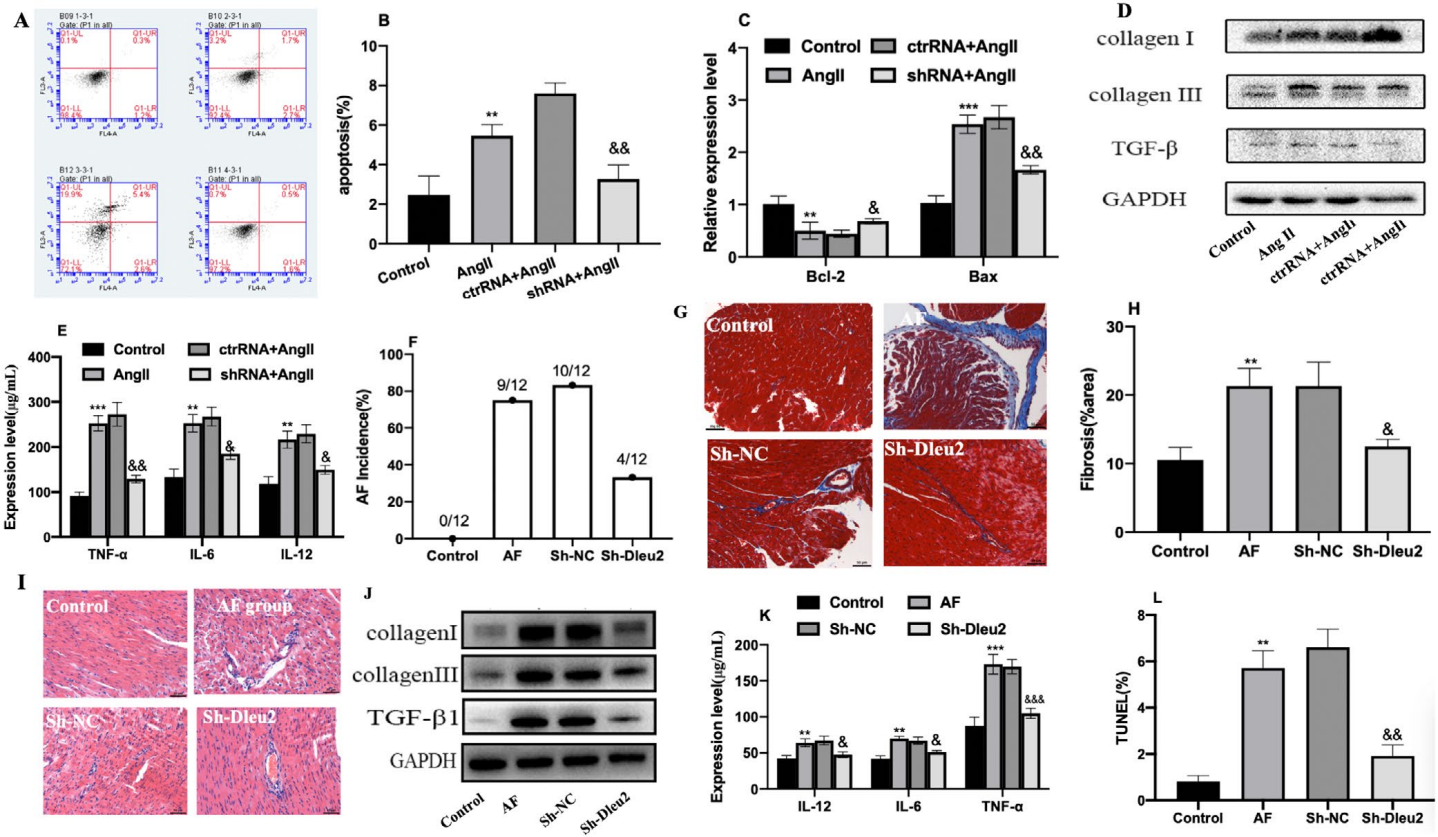
Function of LncRNA Dleu2 on atrial remodelling and AF susceptibility. (A) The cardiomyocyte apoptosis flow cytometry image. (B) Statistical graph of the flow‐cytometric analysis (*n* = 3). (C) Bcl‐2 and Bax mRNA expression levels (*n* = 6). (D) TGF‐β, Collagen I and collagen III protein expression levels. (E) TNF‐α, IL‐12 and IL‐6 expression levels detected with ELISA (*n* = 12). **p < 0.01, ***p < 0.001 versus Control; ^&^ *p* < 0.05, ^&&^ *p* < 0.01 versus ctrRNA + Ang II group. (F) AF incidence (*n* = 12). (G) Representative Masson‐staining image. (H) Statistical graph of Masson staining (*n* = 3). (I) Representative H&E staining image. (J) TGF‐β, Collagen I and collagen III protein expression levels. (K) TNF‐α, IL‐12 and IL‐6 expression levels as measured with ELISA (*n* = 12). (L) Statistical graph of TUNEL staining (*n* = 3). **p < 0.01, ***p < 0.001 versus Control; ^&^ *p* < 0.05, ^&&^ *p* < 0.01, ^&&&^ *p* < 0.001 versus Sh‐NC.

To investigate the functional consequences of LncRNA Dleu2 on AF development, we induced atrial cardiomyocyte remodelling with Ang II and transfected them with Sh‐Dleu2 adenovirus vector to silence LncRNA Dleu2 expression. We found that LncRNA Dleu2 expression was increased in Ang II‐induced atrial cardiomyocytes, and Sh‐Dleu2 successfully silenced LncRNA Dleu2 expression. Three LncRNA Dleu2 specific siRNAs (Sh‐Dleu) were designed to silence LncRNA Dleu2 expression, and two siRNAs successfully silenced LncRNA Dleu2 expression, then we selected the siRNA with the best silencing effect for the subsequent functional study (Figure S1A,B). The proportion of cardiomyocyte apoptosis was significantly augmented (Figure 4A,B); Bcl‐2 expression rose and Bax expression declined (Figure 4C); collagen I and collagen III expression were increased (Figure 4D); and IL‐12, IL‐6 and TNF‐α expression were elevated in the Ang II group (Figure 4E). Notably, these changes were significantly circumvented by downregulation of LncRNA Dleu2 expression (Figure 4A–E).

The AF and atrial‐remodelling animal models were established using continuous injection of Ang II, and an adenovirus vector carrying Sh‐Dleu2 was transduced to silence the expression of LncRNA Dleu2 via tail vein injection. Our results showed that intravenous injection of Sh‐Dleu2 also inhibited LncRNA Dleu2 expression in the atrial tissues in vivo (Figure S1C,D). The AF incidence was enhanced in the AF model group (Figure 4F); atrial fibrosis statistically increased (Figure 4G,H); atrial myocardial cell morphology was disordered, with inflammatory infiltration (Figure 4I); collagen I, collagen III and TGF‐β protein expression rose significantly (Figure 4J); IL‐12, IL‐6 and TNF‐α expression were augmented (Figure 4K); the percentage of myocardial cell apoptosis significantly increased (Figure 4L, Figure S1E); and Bax expression was increased, while Bcl‐2 expression was declined in the AF model group (Figure S1F). These changes were then significantly prevented by downregulation of LncRNA Dleu2 expression (Figure 4F–L, Figure S1E,F).

### 3.5 Target Screening of LncRNA Dleu2

To further explore the mechanism of LncRNA Dleu2 action on AF occurrence, we constructed adenovirus vectors carrying a LncRNA Dleu2 sequence and transfected it into atrial cardiomyocytes. The fluorescence proportion rate of cardiomyocytes was above 80% (Figure S2A), and LncRNA Dleu2 expression was significantly enhanced in the overexpression group, indicating that intracellular transfection was effective (Figure S2B). The differentially expressed molecules regulated by LncRNA Dleu2 were then screened with transcriptomic sequencing, and the selected DEGs were analysed by applying bioinformatics (cluster analysis), providing a DEG cluster diagram (Figure S2C). The DEGs and their statistical significances between the two groups are shown in a volcano map (Figure S2D). The GO annotation, which included biological processes, molecular functions and cellular components, is shown in Figure S2E; and the KEGG pathway‐annotation diagram (Figure S2F), KEGG‐classification diagram of DEGs (Figure S2G), KEGG‐enrichment histogram (Figure S2H) and visualised protein interaction network diagram of DEGs are shown (Figure S2I).

### 3.6 LncRNA Dleu2 Directly Binds the Nr4a1 Protein

**FIGURE 5:**
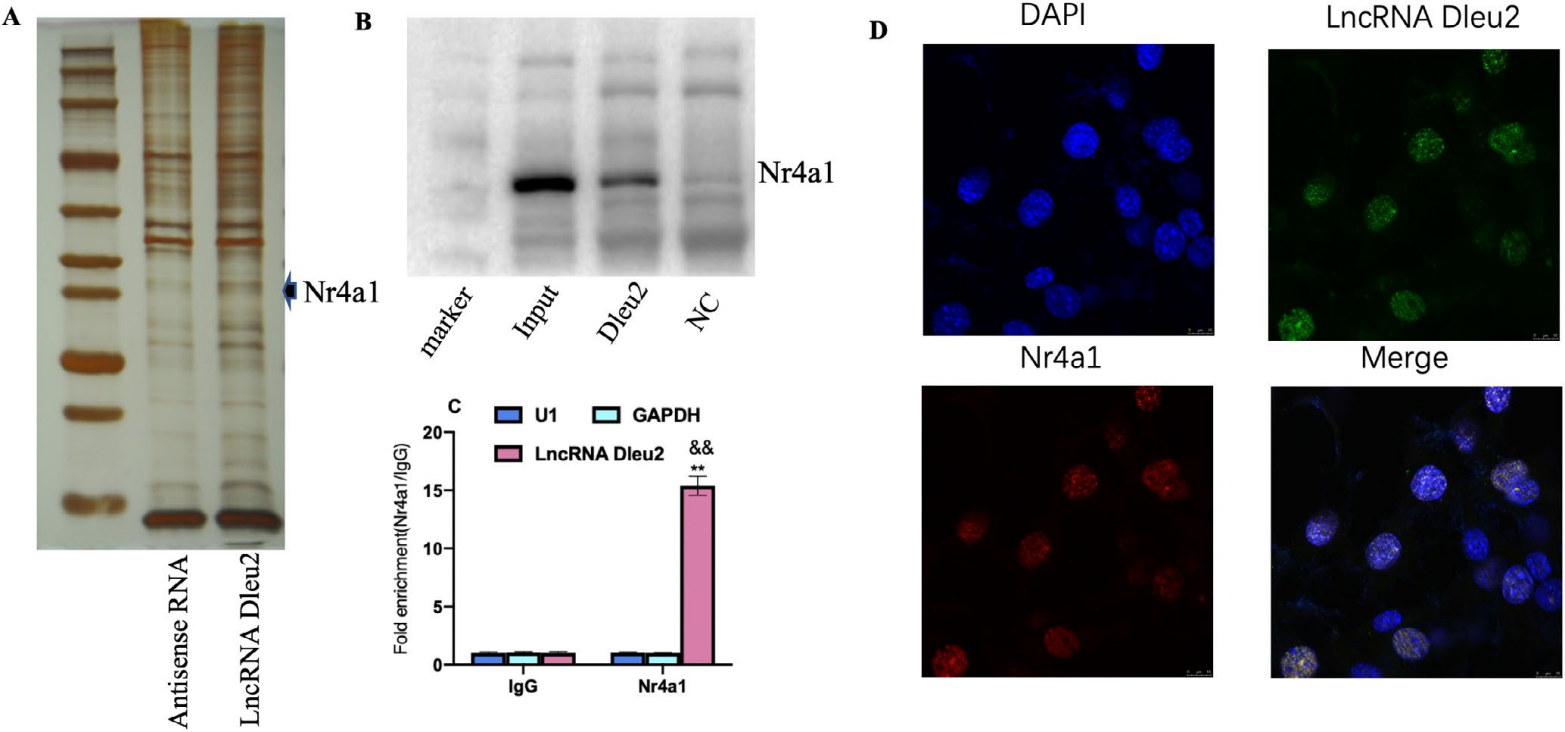
Screening and identification of the direct targets of LncRNA Dleu2. (A) Silver‐staining protein results of RNA pull‐down. (B) Immunoblotting detection of LncRNA Dleu2 specific‐binding protein. (C) QPCR analysis of the indicated RNAs expression level in the RIP assay (*n* = 3). (D) Co‐localization analysis of LncRNA Dleu2 and Nr4a1 using FISH and immunofluorescence. DAPI, 4′,6‐diamidino‐2‐phenylindole. **p < 0.01 versus U1; ^&&^ *p* < 0.01 versus GAPDH.

We obtained a LncRNA Dleu2 direct‐binding protein by RNA pull‐down and further analysed it by protein mass spectrometry, demonstrating the protein by silver staining (Figure 5A). We identified Nr4a1 as the LncRNA Dleu2‐binding protein by independent immunoblotting in an RNA pull‐down experiment (Figure 5B), and subsequently verified the specificity of its interaction with LncRNA Dleu2 by RIP (Figure 5C). Moreover, LncRNA Dleu2 co‐localised with Nr4a1 in the nucleus using RNA FISH followed by immunofluorescence (Figure 5D).

### 3.7 Intervention Nr4a1 Expression Regulated Atrial Remodelling and AF Susceptibility

**FIGURE 6:**
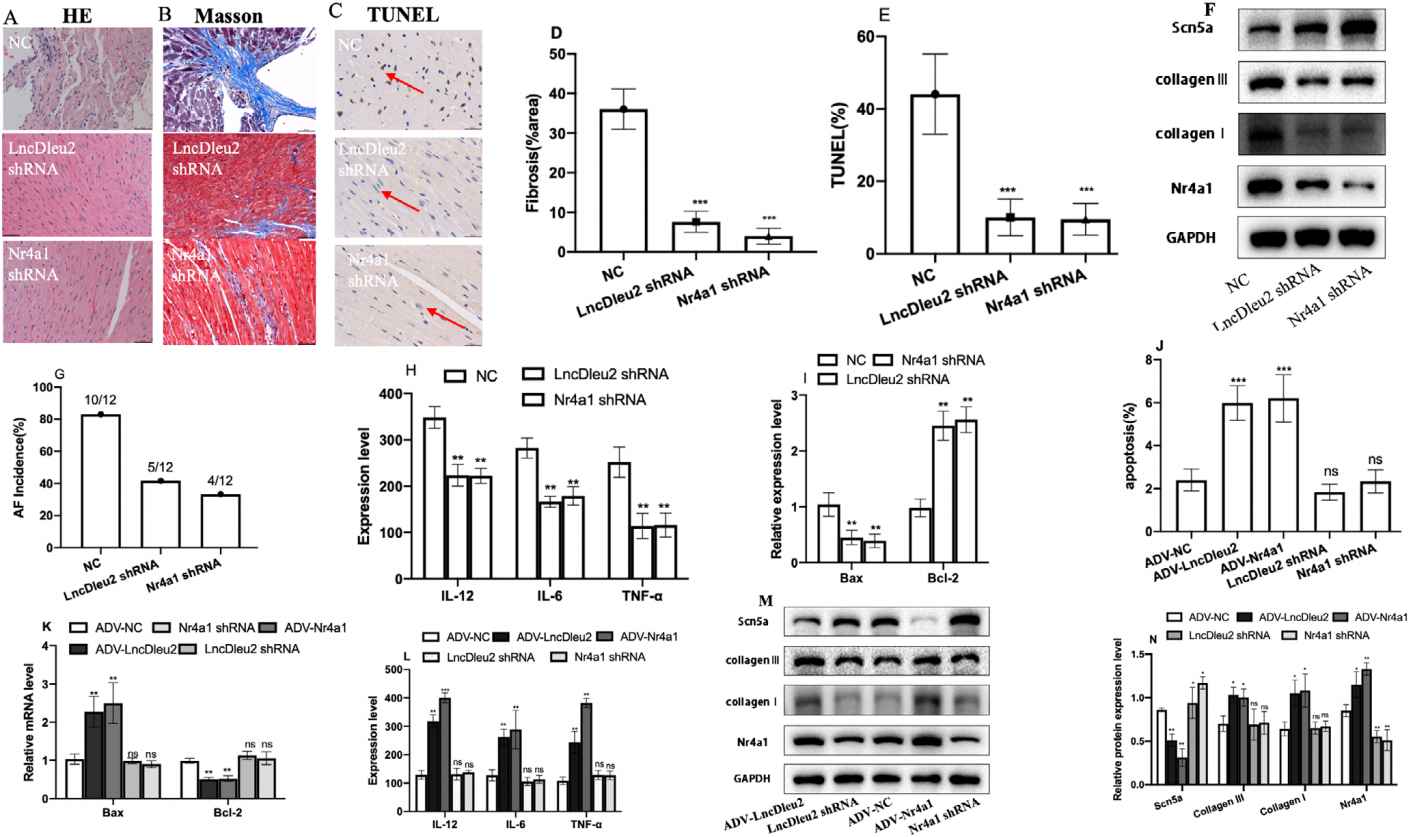
Biological function of Nr4a1 on AF occurrence and atrial remodelling. (A) Representative H&E staining image. (B) Representative Masson‐staining image. (C) Representative TUNEL staining image, apoptotic cells indicated by red arrow. (D) Statistical graph of Masson staining (*n* = 3). (E) Apoptotic rate with TUNEL staining (*n* = 3). (F) Protein expression of Scn5a, collagen I, collagen III and Nr4a1. (G) AF incidence rate (*n* = 12). (H) TNF‐α, IL‐6 and IL‐12 expression (*n* = 12). (I) Expression level of Bax and Bcl‐2 mRNA (*n* = 6). **p < 0.01, ***p < 0.001 versus NC, NC represents the AF + sh‐NC, LncDleu2 shRNA represents AF + sh‐LncRNA Dleu2, Nr4a1 shRNA represents AF + Sh‐Nr4a1. (J) Cardiomyocyte apoptosis rate detected with flow cytometry (*n* = 3). (K) Bax and Bcl‐2 expression levels (*n* = 6). (L) TNF‐α, IL‐6 and IL‐12 expression as detected with ELISA (*n* = 12). (M) Protein expression of Scn5a, collagen I, collagen III and Nr4a1. (N) Quantitative statistical graph of Scn5a, collagen I, collagen III and Nr4a1 protein expression (*n* = 3). **p < 0.01, ***p < 0.001 versus ADV‐NC; ns, not significant.

To further define the biologic function of Nr4a1 on AF occurrence and atrial remodelling, and the relationship between Nr4a and LncRNA Dleu2, we constructed an adenovirus vector carrying Sh‐Nr4a1 and injected it into mice of the AF model through the tail vein to interfere with Nr4a1 expression. Downregulation of Nr4a1 expression in the AF model inhibited atrial inflammatory response and improved myocardial cell structural disorder (Figure 6A); improved atrial fibrosis (Figure 6B,D); reduced the percentage of myocardial cell apoptosis (Figure 6C,E); inhibited collagen I and collagen III expression and promoted SCN5A expression; inhibited Nr4a1 protein expression (Figure 6F); reduced AF incidence (Figure 6G); diminished the IL‐12, IL‐6 and TNF‐α level (Figure 6H, Figure S6B,E); inhibited Bax expression and promoted Bcl‐2 expression (Figure 6I); inhibited angiogenesis and cardiomyocyte hypertrophy(Figure S6). The above results suggested that downregulation of Nr4a1 expression inhibited atrial remodelling and reduced AF susceptibility. In addition, the biological functions upon downregulation of Nr4a1 were comparable to those of LncRNA Dleu2 (Figure 6A–I).

We subsequently further interfered with the expression of Nr4a1 and LncRNA Dleu2 in atrial cardiomyocytes. Overexpression of LncRNA and Nr4a1 both promoted cardiomyocyte apoptosis (Figure 6J, Figure S3); promoted Bax expression and inhibited Bcl‐2 expression (Figure 6K); increased IL‐12, IL‐6 and TNF‐α expression (Figure 6L); and promoted collagen I and collagen III expression (Figure 6M,N, Figure S4B). However, downregulation of LncRNA Dleu2 and Nr4a1 had no effect on the aforementioned biological indices (Figure 6J–N).

Similarly, overexpression of LncRNA Dleu2 and Nr4a1 inhibited SCN5A protein expression, and downregulation of LncRNA Dleu2 and Nr4a1 promoted SCN5A protein expression. The expression of Nr4a1 was elevated irrespective of LncRNA Dleu2 or Nr4a1 overexpression, while Nr4a1 expression was reduced when intervention with LncRNA Dleu2 or Nr4a1 was performed (Figure 6J–N).

### 3.8 LncRNA Dleu2 Exerts Biological Functions Through Nr4a1

**FIGURE 7:**
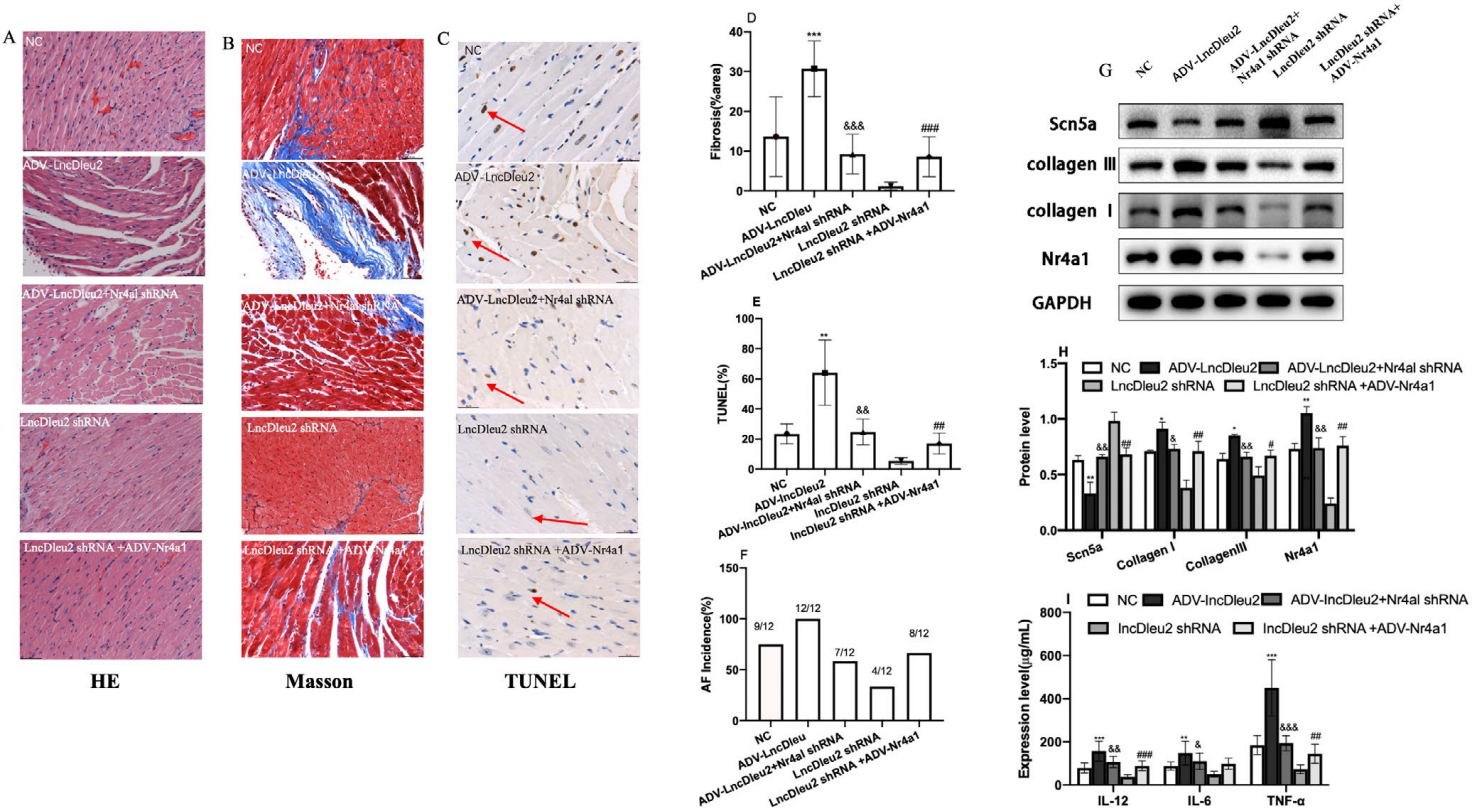
Intervention Nr4a1 expression reversed the biological function of LncRNA Dleu2 in vivo. (A) Representative H&E‐staining image. (B) Representative Masson‐staining image. (C) Representative TUNEL staining image, apoptotic cells indicated by red arrow. (D) Statistical graph of Masson staining (*n* = 3). (E) Apoptotic rates with TUNEL staining (*n* = 3). (F) AF incidence rate (*n* = 12). (G, H) Protein expression of Scn5a, collagen I, collagen III and Nr4a1 (*n* = 3). (I) TNF‐α, IL‐6 and IL‐12 levels (*n* = 12). *p < 0.05, **p < 0.01, ***p < 0.001 versus NC; ^&^ *p* < 0.05, ^&&^ *p* < 0.01, ^&&&^ *p* < 0.001 versus ADV‐lncDleu2; ^#^ *p* < 0.05, ^##^ *p* < 0.01, ^###^ *p* < 0.001 versus lncDleu2 shRNA.

A rescue experiment was conducted to confirm that LncRNA Dleu2 exerts biological functions through Nr4a1. Firstly, overexpression of LncRNA Dleu2 aggravated the disorder of atrial structure (Figure 7A); promoted atrial fibrosis (Figure 7B,D); promoted myocardial cell apoptosis (Figure 7C,E); increased AF incidence (Figure 7F); promoted collagen I and collagen III protein expression; inhibited SCN5A expression (Figure 7G,H); and elevated IL‐12, IL‐6 and TNF‐α expression levels and CD45 (Figure 7I, Figure S7C,E). Improved the angiogenesis and cardiomyocyte hypertrophy (Figure S7). Downregulation of Nr4a1 expression circumvented these changes (Figure 7A–I), and overexpression of Nr4a1 reversed the protective effects of LncRNA Dleu2 downregulation on AF occurrence and atrial remodelling (Figure 7A–I).

**FIGURE 8:**
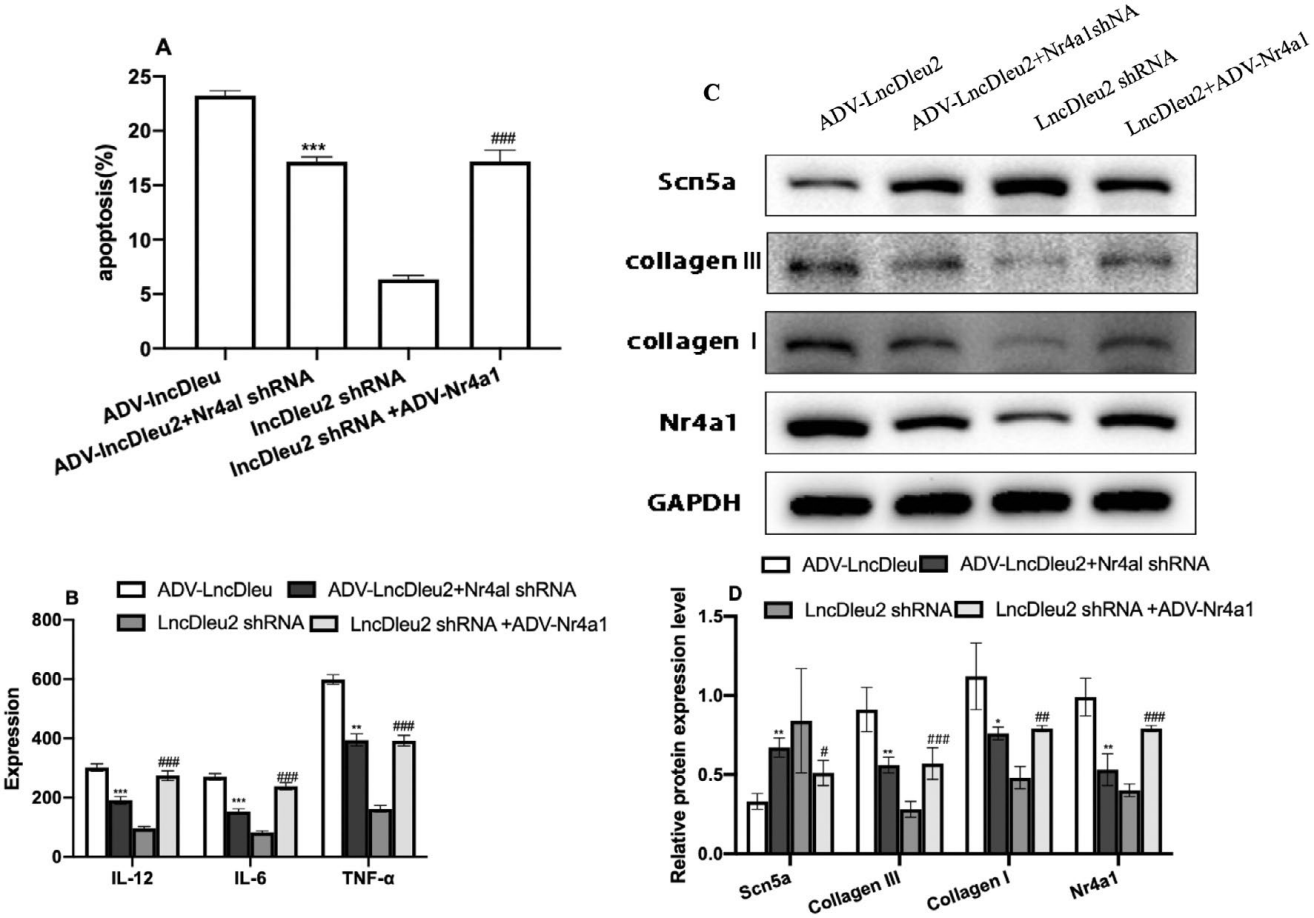
Intervention Nr4a1 expression reversed cardiomyocyte apoptosis and fibrosis of LncRNA Dleu2 in vitro. (A) Cardiomyocyte apoptosis rate detected with flow cytometry (*n* = 3). (B) The TNF‐α, IL‐6 and IL‐12 levels (*n* = 12). (C) Scn5a, collagen I, collagen III and Nr4a1 protein expression. (D) Quantitative statistical graph of Scn5a, collagen I, collagen III and Nr4a1 protein levels (*n* = 3). *p < 0.05, **p < 0.01, ***p < 0.001 versus ADV‐LncDleu2; ^##^ *p* < 0.01, ^###^ *p* < 0.001 versus lncDleu2 shRNA.

We also performed a rescue experiment using the in vitro model to confirm whether LncRNA Dleu2 regulated cardiomyocyte apoptosis and fibrosis via Nr4a1. Our results revealed that downregulation of Nr4a1 expression could reverse the effect of LncRNADleu2 on cardiomyocyte apoptosis (Figure 8A, Figure S4), inflammatory response (Figure 8B), and fibrosis (Figure 8C,D). Similarly, overexpression of Nr4a1 reversed the protective effects of LncRNADleu2 downregulation on cardiomyocyte apoptosis, inflammatory response and fibrosis (Figure 8A–D).

## 4 Discussion

The present study aimed to screen the differentially expressed LncRNA and explored the role of these LncRNAs and its underlying molecular mechanism in AF occurrence, and also analysed the relationship between these differentially expressed LncRNAs and clinical indications such as atrial fibrosis and recurrence after RFCA in AF. We found that LncRNA Dleu2 expression was increased in AF compared with sinus rhythm in the atrial tissue and plasma, and LncRNA Dleu2 expression was positively correlated with left atrial fibrosis and inflammatory response, and could be served as a biomarker for atrial remodelling. Furthermore, LncRNA Dleu2 is involved in regulating AF susceptibility and atrial structural remodelling by directly binding to the Nr4a1 protein.

Atrial remodelling, driven by abnormal gene expression under various adverse pathological conditions, is the principal pathological mechanism of AF development [23]. Aberrant gene expression leads to abnormally coded protein or noncoding RNA expression [24, 25]. Noncoding RNA can serve as a molecular biomarker for the prognosis of AF [26, 27]. Multiple differentially expressed LncRNAs in AF were screened by RNA sequencing, bioinformatics analysis and other methods; and functional studies confirmed that LncRNAs participate in AF occurrence by regulating atrial structural and electrical remodelling [28, 29]. However, the function of LncRNAs in AF development remains to be explored, particularly the association of LncRNAs with atrial fibrosis and inflammatory response. To further explore the role of LncRNA related to fibrosis and inflammation in the occurrence of AF, we constructed two AF animal models and screened differentially expressed LncRNA in the atrial by RNA sequencing. The results demonstrated that 6 LncRNAs, including LncRNA Olfr56, LncRNA Dleu2, A330023F24Rik, 6030408B16Rik, Gm37711 and LncRNA MEG3, showed significant differences in their expression in the atrial tissue of the mouse AF model. Furthermore, bioinformatics analysis showed that only LncRNA Dleu2 and LncRNA MEG3 were highly conserved across species. Current studies have shown that LncRNA MEG3 participates in the occurrence and progression of various cardiovascular diseases [30]. For example, prophylactic inhibition of LncRNA MEG3 expression in the early stage of cardiac remodelling can alleviate cardiac fibrosis and improve cardiac diastolic function [30] Down‐regulating the expression of LncRNA MEG3 can inhibit ischemia–reperfusion‐induced cardiomyocyte apoptosis and exert its function through the miR‐7‐5p/PARP1 pathway [31]. LncRNA MEG3 promotes apoptosis by directly binding to FUS protein and can be regulated by P53 protein [32]. Our previous studies demonstrated LncRNA MEG3 was associated with atrial fibrosis and recurrence post ablation in AF. However, there are few relevant studies on LncRNA Dleu2 in cardiovascular diseases. Studies showed that knockdown of LncRNA Dleu2 suppresses idiopathic pulmonary fibrosis by regulating the microRNA‐369‐3p/TRIM2 axis [33] and Dleu2 modulates proliferation, migration and invasion of vascular smooth muscle cells contributed to atherosclerosis [34], which suggested aberrant expression of LncRNA Dleu2 may be related to the regulatory process of fibrosis or inflammation that contributed to AF development.

Atrial fibrosis is the primary pathological manifestation of atrial structural remodelling, and correlates positively with the progression and prognosis of AF. The chief signal for the transformation of paroxysmal AF to non‐paroxysmal AF is the deterioration of atrial remodelling [35]. Investigators have revealed that LncRNAs participated in regulating the process of atrial fibrosis [16], and can be used as a biomarker of cardiac fibrosis [36, 37, 38]. The Kcnq10t1 gene regulates myocardial fibrosis via the TGF‐β1 signalling pathway [37] and GAS5 affects cellular fibrosis and apoptosis through caspsae‐3 [37]. LncRNA NEAT1 is related to atrial fibrosis and regulates atrial remodelling by affecting fibroblast proliferation and collagen accumulation in AF [38]. Nevertheless, whether LncRNAs related to atrial fibrosis play a more important role in AF development remains to be clarified. The degree of left atrial fibrosis was positively correlated with the prognosis of AF, especially the recurrence of AF after ablation [4]. Screening LncRNAs, which are associated with atrial fibrosis, has important clinical significance for predicting the extent of atrial fibrosis. They can also become a biomarker for screening persistent AF patients who are suitable for RFCA to reduce the recurrence rate. Our study demonstrated that LncRNA Dleu2 expression was increased in persistent AF compared with paroxysmal AF, and was positively associated with left atrial fibrosis and the recurrence rate after AF ablation. This suggests that LncRNA Dleu2 may be used as a fibrosis biomarker for preoperative evaluation; for example, treating patients with persistent AF and mild left atrial fibrosis with ablation will improve the success rate, and avoiding ablation therapy for AF with severe left atrial fibrosis will reduce unnecessary medical expenses. Interestingly, the present study showed that circulating LncRNA Dleu2 was restored after RFCA in persistent AF, and the ROC curve also demonstrated that LncRNA Dleu2 has predictive ability with respect to recurrence after AF ablation. This further suggests that LncRNA Dleu2 can serve as a novel molecular model to predict AF recurrence after RFCA. More critically, our results demonstrated that LncRNA Dleu2 knockdown significantly reduced atrial fibrosis and attenuated cardiac hypertrophy, thereby inhibiting adverse atrial remodelling and decreasing AF susceptibility. These findings position LncRNA Dleu2 as a key regulator in AF pathogenesis and suggest it as a promising molecular target for therapeutic strategies.

Interstitial transformation of endocardial cells [39], cardiomyocyte apoptosis and fibroblast proliferation in the myocardium [16], and abnormal epicardial fat secretion factors or infiltration contribute to atrial remodelling [40], and then promote the development and progression of AF. However, studies on the LncRNAs role in atrial remodelling have mainly focused on regulating fibroblast proliferation and fibrosis [16, 17]. There are few reports concerning LncRNA regulation of myocardial apoptosis. More importantly, apoptosis of atrial myocardium is an early manifestation of atrial remodelling. Exploring the process of myocardial apoptosis is helpful to identify and intervene in the process of atrial remodelling, reducing the onset and delaying the progression of AF. Meanwhile, studies reported that the atrial inflammatory response acts as a crucial role in the AF occurrence and progression, and can be used as a biomarker for the onset, deterioration, and prognosis of AF [41]. The present study demonstrated that LncRNA Dleu2 is involved in regulating atrial remodelling and AF development, and exerts its biological function by regulating myocardial cells apoptosis and inflammatory response, which further expands the spectrum of the role of LncRNAs in AF. However, it is worth noting that intervention in LncRNA Dleu2 expression under normal conditions has no effect on atrial remodelling, suggesting that elevated LncRNA Dleu2 expression may be a pathogenic triggering biomarker in atrial remodelling and AF development. This would create a theoretical foundation for LncRNA Dleu2 to become a molecular target in AF therapy. Meanwhile, in the in vivo apoptosis experiment detection of this study, we only detected the total myocardial cells apoptosis and did not specifically detect the apoptosis of cardiomyocytes. But in the in vitro experiment, the specific apoptosis of cardiomyocytes was detected. We know that in vitro findings are complementary but do not fully recapitulate in vivo complexity. Further in vivo experiments are needed to determine the role of cardiomyocyte apoptosis in atrial remodelling.

The renin‐angiotensin‐aldosterone system (RAAS), which is overactivated by various pathological adverse factors, may promote fibroblast proliferation, myocardial hypertrophy and apoptosis, and contribute to atrial remodelling and induce AF [42]. The animal model used to study the pathogenesis of AF mainly includes drugs and rapid atrial pacing [43, 44]. The AF animal model constructed by Ang II is the most commonly utilised animal model to explore atrial remodelling in AF, and is also suitable for simulating the pathological process of atrial remodelling in clinical AF [45]. This study also utilised Ang II to construct an AF animal model and explore the role of LncRNA Dleu2 in AF occurrence, which provided more reliable evidence for future clinical research of LncRNA Dleu2.

LncRNAs exert their biological functions primarily as competitive endogenous RNA through miRNAs so as to regulate target gene expression in cardiovascular diseases [16, 17, 46]. Although LncRNAs can exert biological functions through a direct binding protein in tumours [47], they have rarely been reported in cardiovascular diseases. This study elucidates the protein interactome of LncRNA Dleu2 through RNA pull‐down assays coupled with mass spectrometry, identifying Nr4a1 as its direct binding partner. Functional validation confirmed that the biological functions of LncRNA Dleu2 are dependent on Nr4a1, thereby establishing a critical regulatory axis between LncRNA and protein signalling. Nr4a1, a member of the nuclear receptor superfamily related to steroid, thyroid and retinoid receptors, has been implicated in diverse biological processes, including T cell regulation, inflammatory responses, apoptosis and fibrotic pathways [48, 49, 50]. Previous studies have demonstrated that abnormal expression of Nr4a1 is implicated in regulating cardiac fibrosis and apoptosis [51, 52]. In this study, we first demonstrated that modulating Nr4a1 expression improves atrial remodelling and reduces susceptibility to AF. Moreover, our findings revealed that altering Nr4a1 expression regulates the expression of SCN5A, which has been confirmed to influence atrial electrical remodelling and contribute to AF development [53, 54]. These results suggest that LncRNA Dleu2 may promote AF pathogenesis by modulating atrial electrical remodelling, a potential mechanism that warrants further investigation in future studies.

It is important to note the limitations of the present study. First, the clinical sample size was small, and it is therefore necessary to conduct multi‐centre and prospective studies in the future to verify the association between LncRNA Dleu2 and atrial fibrosis, as well as recurrence after ablation. Second, we only assessed inflammatory factor expression levels, and additional experiments should be conducted in the future to demonstrate the correlation between LncRNA Dleu2 and inflammatory response during AF development. Thirdly, we herein demonstrated that intervention in LncRNA Dleu2 expression affected SCN5A expression, which has been demonstrated to contribute to AF occurrence [53]; however, more experiments are needed to confirm the correlation between LncRNA Dleu2 and SCN5A. Finally, the cardiomyocyte apoptosis was only detected in the in vitro study with flow cytometry, and the TUNEL assays revealed myocardial cell apoptosis in atrial tissues; this approach does not specifically isolate cardiomyocyte apoptosis. Given the cellular heterogeneity of cardiac tissue, apoptosis observed in our in vivo experiments may also involve non‐cardiomyocyte populations. Future studies will simultaneously stain cardiomyocytes with cardiomyocyte‐specific protein antibodies to determine the role of cardiomyocyte apoptosis in atrial remodelling.

## 5 Conclusions

We investigated the association between LncRNA Dleu2 and clinical indices in AF, and the potential role and mechanism of LncRNA Dleu2 on AF development. We found the abnormal expression of LncRNA Dleu2 was associated with atrial fibrosis and recurrence after AF ablation, and this contributed to atrial remodelling by regulating myocardial cell apoptosis and inflammatory response via directly binding Nr4a1 protein. This study provides a theoretical foundation for LncRNA Dleu2 serving as a biomarker for atrial fibrosis and recurrence prediction after AF ablation. We postulate that modalities that target LncRNA Dleu2 may constitute a valuable therapeutic strategy for AF. But further studies using cardiomyocyte‐specific models are warranted to dissect the precise contribution of cardiomyocyte apoptosis to AF pathogenesis.

## Author Contributions

**Feiyu Wei:** conceptualization (equal), funding acquisition (equal), methodology (lead), writing – original draft (lead). **Yazhe Ma:** methodology (equal). **Hong Xiang:** methodology (equal). **Xi Zhang:** conceptualization (supporting), funding acquisition (equal), supervision (supporting), writing – review and editing (supporting). **Jie Fan:** conceptualization (lead), funding acquisition (lead), project administration (equal), supervision (lead), writing – review and editing (lead).

## Conflicts of Interest

The authors declare no conflicts of interest.

## Supporting information


Data S1.

## Data Availability

The datasets used and/or analysed during the current study are available from the corresponding author on reasonable request.
