# Supplementary material for: LncRNA Dleu2 Serve as a Novel Biomarker for Ablation Recurrence and Promote Atrial Remodelling by Targeting Nr4a1 in Atrial Fibrillation
Source: J Cell Mol Med. 2025 Jun 12;29(11):e70618. doi: 10.1111/jcmm.70618 (PMC12162266; doi:10.1111/jcmm.70618)
Supplement: Supplementary file 1 — Data S1. [file JCMM-29-e70618-s001.docx]

| **Table S1**: Baseline characteristics of the included AF and Non-AF (Values are given as mean ± SD or n (%)) | | | | | | | | |
| --- | --- | --- | --- | --- | --- | --- | --- | --- |
| Variables | Non-AF(n=80) | | Paro-AF(n=40) | Pers-AF(n=40) | | | | *P-*value |
| Age(years) | 62±7 | 65±8 | | | 64±7 | | 0.09 | |
| Gender (Female) | 46/80(58%) | 22/40(55%) | | | | 26/40(65%) | 0.63 | |
| Left ventricular ejection fraction (%) | 62±5 | 64±6 | | | | 63±7 | 0.20 | |
| Left atrial diameter (mm) | 32±5 | 36±5 | | | | 40±5 | <0.001 | |
| Left ventricle end-diastolic diameter (mm) | 43±7 | 45±5 | | | | 44±7 | 0.28 | |
| BMI (kg/m^2^) | 24.1±3.1 | 23.5±2.9 | | | | 24.4±2.7 | 0.40 | |
| Type2 DM | 20/80(25%) | 12/40(30%) | | | | 11/40(28%) | 0.07 | |
| Hyperlipemia | 13/80(16%) | 9/40(23%) | | | | 10/40(25%) | 0.48 | |
| Stroke | 0/80 | 2/40(5%) | | | | 5/40(13%) | 0.24^*^ | |
| Hypertension | 26/80(33%) | 19/40(48%) | | | | 17/40(43%) | 0.24 | |
| Medication history (%) |  |  | | | |  |  | |
| β-blockers | 21/80(26%) | 15/40(38%) | | | | 11/40(28%) | 0.42 | |
| Amiodarone | 0/80(0) | 3/40(8%) | | | | 9/40(23%) | 0.12* | |
| propafenone | 0/80(0) | 7/40(18%) | | | | 2/40(5%) | 0.15* | |
| ACE I or ARB | 20/80(25%) | 10/40(25%) | | | | 13/40(33%) | 0.65 | |
| Calcium antagonist | 24/80(30%) | 14/40(35%) | | | | 8/40(20%) | 0.31 | |
| Left atrial low voltage area degree | |  | | | |  |  | |
| 0% to 10% |  | 4^#^ | | | | 32(80%) |  | |
| 10% to 20% |  |  | | | | 6(15%) |  | |
| >30% |  |  | | | | 2(5%) |  | |
| Low voltage area distribution | |  | | | |  |  | |
| Anterior wall | / | 2/40 | | | | 11/40 |  | |
| Posterior wall | / | 2/40 | | | | 14/40 |  |  |
| Roof wall | / | / | | | | 3/40 |  |  |
| Septal | / | / | | | | 5/40 |  |  |
| Ablation strategy |  |  | | | |  |  | |
| pulmonary vein isolation | / | 40/40 | | | | 40/40 |  | |
| Roof line | / | 0 | | | | 38/40 |  |  |
| Isthmus line of Mitral valve | / | 0 | | | | 4/40 |  |  |
| Cavo-tricuspid isthmus; |  | 2/40 | | | | 33/40 |  |  |
| LA anterior line |  | 1/40 | | | | 7/40 |  |  |
| LA posterior line | / | 2/40 | | | | 38/40 |  |  |
| Superior vena cava isolation | / | 33/40 | | | | 10/40 |  |  |
| Inflammatory factor(μg/mL) |  |  | | | |  |  | |
| TNF-α | 86±16 | 274±40 | | | | 339±121* | <0.001 | |
| IL-6 | 108±22 | 243±29 | | | | 336±31* | <0.001 | |
| IL-12 | 102±25 | 236±34 | | | | 339±32* | <0.001 | |

BMI: Body mass index, DM: diabetes mellitus, LA: Left atrial. ^*^ represented the statistical difference between the Paro-AF and Pers-AF groups. Paro-AF: Paroxysmal AF, Pers-AF: Persistent AF. *<0.001 *vs* Paro-AF.

| Table S2: Baseline characteristics of the 9 AF and 9 non-AF | | | |
| --- | --- | --- | --- |
| Parameter | Non-AF (n=9) | AF (n=9) | P value |
| Gender (Female) n (%) | 5 (56%) | 6 (67%) | 1 |
| Age(years) | 46±14 | 43±9 | 0.59 |
| Rheumatic heart disease | 4 (44%) | 5 (56%) | 1 |
| Congenital heart disease | 5 (56%) | 4 (44%) | 1 |
| Left atrial diameter (mm) | 44±6 | 47±8 | 0.90 |
| Left ventricular ejection fraction (%) | 59±4 | 62±8 | 0.33 |
| Left ventricle end-diastolic diameter (mm) | 47±8 | 49±6 | 0.56 |

| **Table S3**: Association between the LncRNA Dleu2 and AF onset by logistic regression | | | | |
| --- | --- | --- | --- | --- |
| Model | Odds ratio | 95% Confidence interval | | P value |
| Model 1 | 2.30 | 1.31-3.98 | 0.03 | |
| Model2 | 2.34 | 1.23-4.51 | 0.02 | |

Model 1: unadjusted.

Model 2: adjusted for LAD, Type2 DM.

**Supplementary Material and methods**

**Expanded methods**

**Study populations**

The inclusion criteria of the internal RFCA cohort consisted of patients who were diagnosed with non-valvular AF and who met the indications for radiofrequency catheter ablation in accordance with the 2016 European Society of Cardiology Guidelines for the Management of AF^[1]^. The exclusion criteria were AF patients who were aged <18 years or >80 years; or who were pregnant or manifested severe underlying structural heart disease, serious liver or renal dysfunction, a LA diameter ≥55 mm, or LA or LA appendage thrombosis. All patients signed informed consent forms.

**Animals and treatments**

Male C57BL/6 mice (aged 6–8 weeks, 20-25g) were purchased from SLAC (Shanghai, China). Animal experimentation was conducted in accordance with the Guide for the Care and Use of Laboratory Animals, and all study procedures were approved by the Institutional Animal Care and Use Committee of Kunming University of Science and Technology.

In the first type of AF animal model, 6–8-week-old male C57BL/6 wild-type mice were induced by acetylcholine calcium chloride (Ach and CaCl2). The acetylcholine calcium chloride (60 μg/ml Ach and 10 mg/ml CaCl2, administered at a dosage of 1 ml/kg/d) were continuously injected daily by tail vein for one week, while the control group was injected with 0.9% PBS daily. Surface electrocardiography was monitored every day to record the spontaneous AF rate.

In another type of AF animal model, 6–8-week-old male C57BL/6 wild-type mice were taken and an osmotic-pressure micropump capsule (Alzet, USA) was implanted subcutaneously. The dosage of Ang II was calculated according to mouse weight and Alzet instructions, and Ang II (2000 ng/kg/min) was continuously perfused for three weeks to establish the AF model. Mice were injected with tribromoethyl alcohol intraperitoneally, and their backs disinfected with 75% medical alcohol after anesthesia. A 1-cm-long vertical incision was made in the middle shoulder, and the skin and subcutaneous tissue were separated from the tail using hemostatic forceps. The perfused capsule with osmotic pressure micropump was then injected into the subcutaneously separated area, and the area was sutured with surgical thread and disinfected with iodophor. Surface electrocardiography was monitored every day to record the spontaneous AF rate. Mice were deeply anesthetized via intraperitoneal injection of pentobarbital sodium (100 mg/kg) to ensure complete unconsciousness and absence of pain reflexes. For tissue collection requiring intact morphology (e.g., HE, Masson, TUNEL and immunohistochemistry), euthanasia was performed by cervical dislocation under anesthesia.

**RNA sequencing**

Total RNA was isolated and purified using TRIzol reagent (Invitrogen, Carlsbad, CA, USA) following the manufacturer’s procedure, the purity of each sample was quantified using a NanoDrop ND-1000 (NanoDrop, Wilmington, DE, USA), and RNA integrity was assessed using an Agilent 2100 with RIN number >7.0. Approximately 5 μg of total RNA was used to deplete ribosomal RNA according to the instructions of the Ribo-Zero™ rRNA Removal Kit (Illumina, San Diego, USA). After removing ribosomal RNAs, the remaining RNAs were fragmented into small pieces using divalent cations under high temperature. The cleaved RNA fragments were then reverse-transcribed to create cDNA, which was used to synthesize U-labeled, second-stranded DNAs with E. coli DNA polymerase I, RNase H, and dUTP. An A-base was added to the blunt ends of each strand, preparing them for ligation to the indexed adapters, and each adapter contained a T-base overhang for ligating the adapter to the A-tailed fragmented DNA. Single-or dual-index adapters were ligated to the fragments, and size selection was performed with AMPureXP beads. After heat-labile UDG-enzyme treatment of the U-labeled second-stranded DNAs, the ligated products were amplified by PCR using the following conditions: initial denaturation at 95°C for 3 min, 8 cycles of denaturation at 98°C for 15 s, annealing at 60°C for 15 s, and extension at 72°C for 30 s; with a final extension at 72°C for 5 min. The average insert size for the final cDNA library was 300 bp (±50 bp). Lastly, we performed paired-end sequencing on an Illumina HiSeq 4000 (LC Bio, China) following the vendor's recommended protocol.

**Flow cytometry**

The atrial cardiomyocytes that reached a growth density of 80% were used to further study. The cells were digested with trypsin without EDTA and collected by centrifugation, and the cellular suspension was added to 5 μL of annexin V FITC and 10 μL of PI, gently mixed, and incubated for 5 min at 4°C in the dark; apoptotic rate was then determined using a flow cytometer (ACCURI C6, BD Biosciences).

**Western blotting**

Total protein was extracted with lysis buffer containing protease inhibitor cocktail, and the protein was transferred to polyvinylidene difluoride membranes and blocked in PBS containing a 5% fat-free milk powder. The corresponding proteins were detected with antibodies against collagen I (1:1000, ab34710, Rabbit, abcam), collagen III (1:1000, BS0549R, Rabbit, bioss), TGF-β (1:1000, BS1316, Rabbit, bioworld), Nr4a1(1:1000, YT3213, Rabbit, immunoway), SCN5A (1:1000, ab300048, Rabbit, abcam) and GAPDH (1:1000, Mab5465, Rabbit, MULTI SCIENCES BIOTECH). We utilized goat anti-rabbit (1:5000, GAR0072, MULTI SCIENCES BIOTECH) or goat anti-mouse (1:5000, GAM007, MULTI SCIENCES BIOTECH). A Bio-Rad gel imager was used to scan and photograph the gels (we have described the detailed protocols previously^[2]^.

**qRT-PCR**

Total RNA was isolated from the plasma and tissue according the manufacturer’s instructions, and cDNA was synthesized from 1.0 mg of total RNA with an anchored-oligo (dT) 15 primer and random primers using a Transcriptor First Strand cDNA Synthesis Kit (Roche, Basel, Switzerland) based on the manufacturer’s instructions; GAPDH was used for normalization. We performed PCR on an ABI 7900 real-time PCR instrument (Applied Biosystems, Foster, CA, United States), and relative RNA expression was determined by applying 2^-△△CT^. The PCR primers we used were as follows.

| Primer sequences for quantitative real-time PCR. | | | |
| --- | --- | --- | --- |
| Target Gene | Forward primer (5’-3’) | | Reverse primer (5’-3’) |
| GAPDH (human) | TGTGGGCATCAATGGATTTGG | ACACCATGTATTCCGGGTCAAT | |
| GAPDH (Mice) | AATGGATTTGGACGCATTGGT | TTTGCACTGGTACGTGTTGAT | |
| Bax (Mice) | AGACAGGGGCCTTTTTGCTAC | AATTCGCCGGAGACACTCG | |
| Bcl-2 (Mice) | GAGCCTGTGAGAGACGTGG | CGAGTCTGTGTATAGCAATCCCA | |
| Collagen I  (Mice) | ATCGGATACTCCTTCCTCATGC | CCAGGGGAGACTAGGGACTG | |
| Collagen III (Mice) | GGCAAGCCTGGACGAAGAG | CCGGTGTACCCTTTTCCCC | |
| LncRNA Olfr56 | CTGGACAGGGATTTGGGACT | ATCCATCTCCCCAGTCTCCT | |
| LncRNA Dleu2 (Mice) | TAAGGAAGCAAGATGGGCCA | GCTGTCCTGGAACTCACTCT | |
| LncRNA Dleu2 (Human) | TCTGACCTGGTTACTCAGTCA | GCTGTCCTGGAACTCACTCT | |
| A330023F24Rik | CTGGACAGGGATTTGGGACT | ATCCATCTCCCCAGTCTCCT | |
| 6030408B16Rik | TCCTGTCTTGCCTGATGCG | AGCCGTGGGAAGCGATG | |
| Gm37711 | ATGATGGAGGGGCAGAGGT | TGGGAAGGGTCAAGGAACAG | |

**RNA immunoprecipitation (RIP)**

The RIP kit (Thermo Fisher Scientific, Waltham, MA, USA) was used in the RIP assay following the manufacturer’s instructions. Briefly, pretreated cardiomyocytes were lysed with RNA lysis buffer and incubated with freshly prepared nuclear separation buffers for 20 min on ice. The lysate was then centrifuged at 2,500 g for 15 min to precipitate the nuclei, and we re-suspended the nuclear precipitation in the freshly prepared RIP buffer. The nuclear precipitate was divided into two parts, at a volume of 500 µL each (for simulation and IP), and we mashed the chromatin 15–20 times using mechanical clipping, and precipitated the nuclear cell membranes and debris at 13,000 rpm for 10 min. The antibody (2–10 µg) generated against the protein of interest was added to the supernatant (6–10 mg) and gently stirred at 4°C for 2 h; protein A/G magnetic beads (40 µL) were then added and the complex gently stirred and incubated at 4°C for 1 h. We washed the unbound substance and centrifuged it at 2,500 rpm for 30 s to precipitate the magnetic beads, removed the supernatant, and re-suspended the magnetic beads in 500 µL of RIP buffer; we cleaned them three times in RIP and once in PBS. The RNA bound to RNA-binding protein (RBP) after immunoprecipitation was purified, and the RNA was extracted according to the RNA extraction method and reverse-transcribed into cDNA for PCR detection.

**RNA pull-down**

LncRNA Dleu2 probe and the control probe were synthesized by Sangon Biotech (Shanghai, China). The cardiomyocytes were lysed with lysis buffer, and the lysed cells were incubated with the LncRNA Dleu2 and control probes. We incubated the cell lysates with streptavidin-coated magnetic beads to pull-down the biotin-conjugated RNA complex, and the beads were rinsed and the RNA complex purified using TRIzol (Takara, Dalian, China). Lastly, we rinsed and eluted RBP complexes and resuspended the magnetic beads with a 1-mL wash buffer. We added 40 μL of 1×SDS loading buffer (including DTT) and treated the complexes at 95°C with boiling water for 5 min. The supernatant was ultimately used for silver staining, SDS-PAGE, or spectrum analysis.

**Fluorescence in-situ hybridization (FISH)**

FISH was used to detect the subcellular localization of LncRNA Dleu2 in cardiomyocytes. The cardiomyocytes were fixed with 4% paraformaldehyde at room temperature for 20–30 min. Digoxin-labeled locked-nucleotide-modified LncRNA Dleu2 probes were diluted with hybridization solution, hybridized overnight at 42°C, and subsequently incubated overnight at 4°C with anti-digoxin monoclonal antibodies. The Nr4a1 was stained with antibodies against Nr4a1（1:1000, YT3213, Immunoway, rabbit, secondary antibody( Donkey anti-rabbit, 1:5000, ab150073, Abcam). We utilized a double-labeled simulated probe for a control group and observed the cells by confocal laser microscopy (DMi8, Leica, Germany).

**LncRNA target gene-screening strategy**

Protein mass spectrometry was first used to determine the peptide chain information for LncRNA Dleu2-binding protein after RNA pull-down, and functional analysis of differentially expressed proteins was conducted applying the Kyoto Encyclopedia of Genes and Genomes (KEGG) enrichment analysis and Gene Ontology (GO) analysis to obtain the functional signaling pathways. Our results showed that MAPK signaling pathway was an important enrichment-signaling pathway for the LncRNA Dleu2-targeting protein. MAPK-signaling pathway activation has been reported to induce cardiac fibrosis ^[3,4]^, and we found that this signaling pathway was related to the synthesis and secretion of aldosterone, an important factor in promoting cardiac fibrosis and a downstream effector of the Renin-angiotensin-aldosterone system^[5]^. We ascertained that Nr4a1 was also involved in the synthesis and secretion of aldosterone. Nr4a1 is a type of solitary nuclear receptor that belongs to the steroid/thyroid/retinoid receptor superfamily. Authors have recently reported that Nr4a1 is involved in the regulation of T cell function, inflammatory response, apoptosis, and fibrosis ^[6-8]^.^.^ We therefore selected Nr4a as the target gene of LncRNA Dleu2 for further study.

Immunohistochemical

We conducted immunohistochemical of the CD34 and CD45 expression to evaluate the angiogenesis and inflammatory infiltration. Tissues were fixed, paraffin-embedded, and stored at -20°C after solidification. 4-µm-thick sections were cut and dried in a 65°C incubator for 6–12 hours to enhance adhesion. The dewaxed slices were then hydrated in ethanol solutions of different concentrations, and then rinsed with distilled water. Incubate with 3% H₂O₂ (37°C, 10 min) to quench peroxidase activity, PBS wash 3 min for 3 times. Heat-induced retrieval in EDTA buffer (pH 9.0, 10 min boiling), followed by natural cooling to room temperature, PBS wash 3 min for 3 times. Overnight incubation in 4℃ refrigerator (Brilliant Violet 510™ anti-mouse CD45 103137, CD34 Monoclonal Antibody (RAM34), FITC, eBioscience™, 11-0341-82), transferred to room temperature equilibrium for 30min, and washed with PBS for 5min. Secondary antibody (PV-6001, goat, ZSBG-BIO) was added, incubated at 37℃ for 30min, and rinsed with PBS for 5min. DAB reaction staining was performed, the reaction progress was observed under microscope, and the reaction was fully rinsed with tap water. and the sections were observed under a fluorescence microscope.

Phalloidin

We conducted Phalloidin staining to evaluate the size of cardiomyocytes in cardiac tissue. The prepared paraffin-embedded tissue block was mounted on a microtome and sectioned into slices with a thickness of approximately 4-6 μm. The sections were then dewaxed by soaking in xylene for a sufficient period to remove the paraffin. Following dewaxing, the sections were hydrated through a graded series of ethanol solutions and rinsed with distilled water. The sections were fixed in PBS containing 3-4% formalin at room temperature for 10-30 minutes. After fixation, the sections were incubated with a working solution of 5 μg/mL (Phalloidin) at room temperature for 30-60 minutes in the dark. The sections were rinsed 2-3 times with PBS for 5 minutes each. The nuclei were counterstained with DAPI for 10 minutes, followed by two additional rinses with PBS. The sections were mounted with a fluorescent mounting medium (composed of a neutral or alkaline buffer mixed with an equal volume of glycerin). A coverslip was applied, and the sections were observed under a fluorescence microscope.

**Expanded Figures and Figure legends**

**
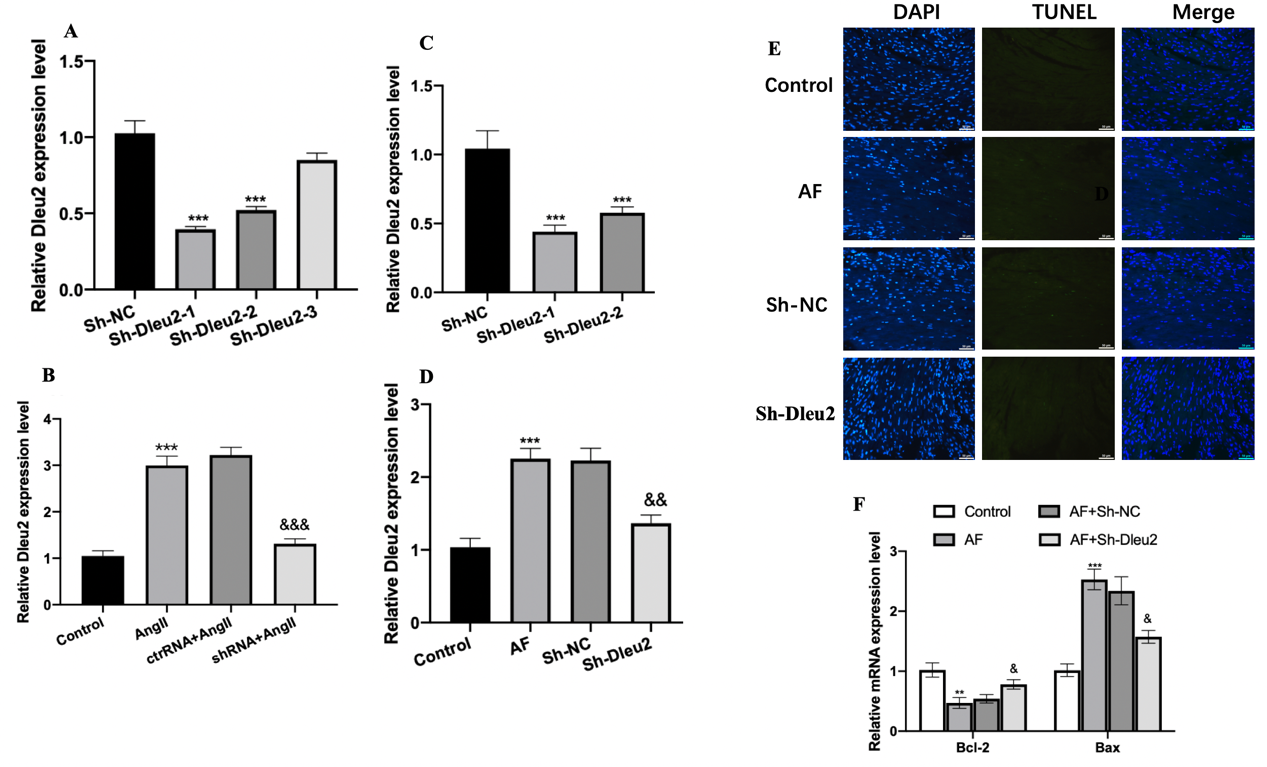
**

**Figure. S1:** Effect of LncRNA Dleu2 on atrial remodeling. **A**: The LncRNA Dleu2 expression level with three LncRNA Dleu2 specific siRNAs in cardiomyocyte. **B**: LncRNA Dleu2 expression levels in cardiomyocytes induced with Ang II. **C**: The LncRNA Dleu2 expression level with two LncRNA Dleu2 specific siRNAs in mouse. **D**: LncRNA Dleu2 expression levels in atrial tissue induced with Ang II. **E**: Representative TUNEL-staining image. **D**: Expression levels of Bcl-2 and Bax. **P<0.01, ***P<0.001 *vs.* Control; ^&^P<0.05, ^&&^P<0.01, ^&&&^P<0.001*vs.* Sh-NC.


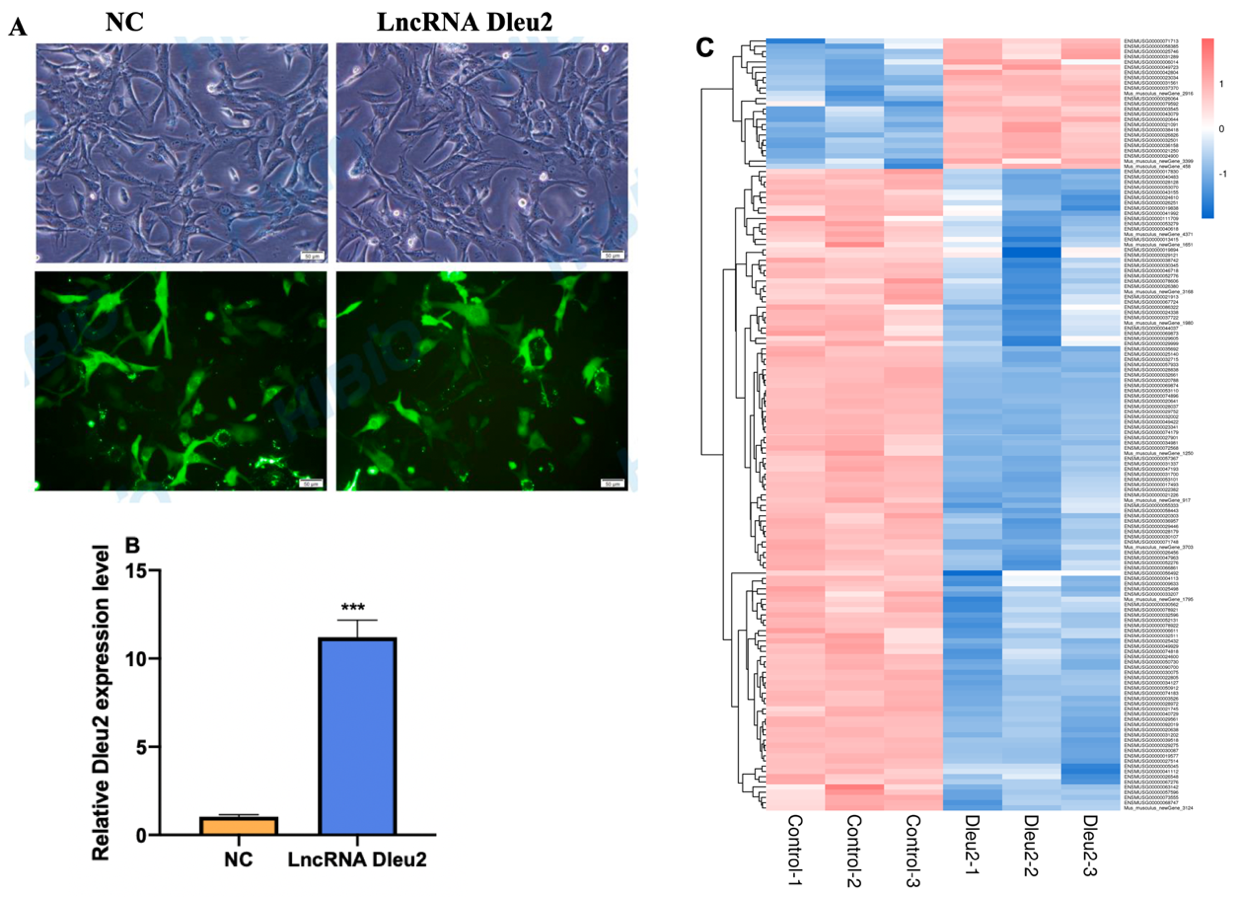

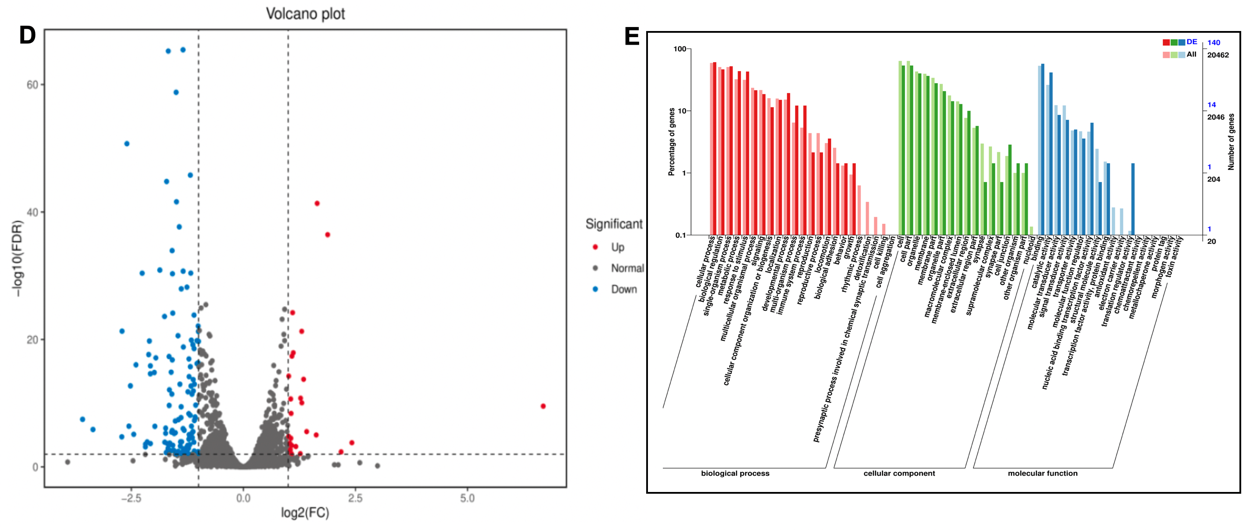

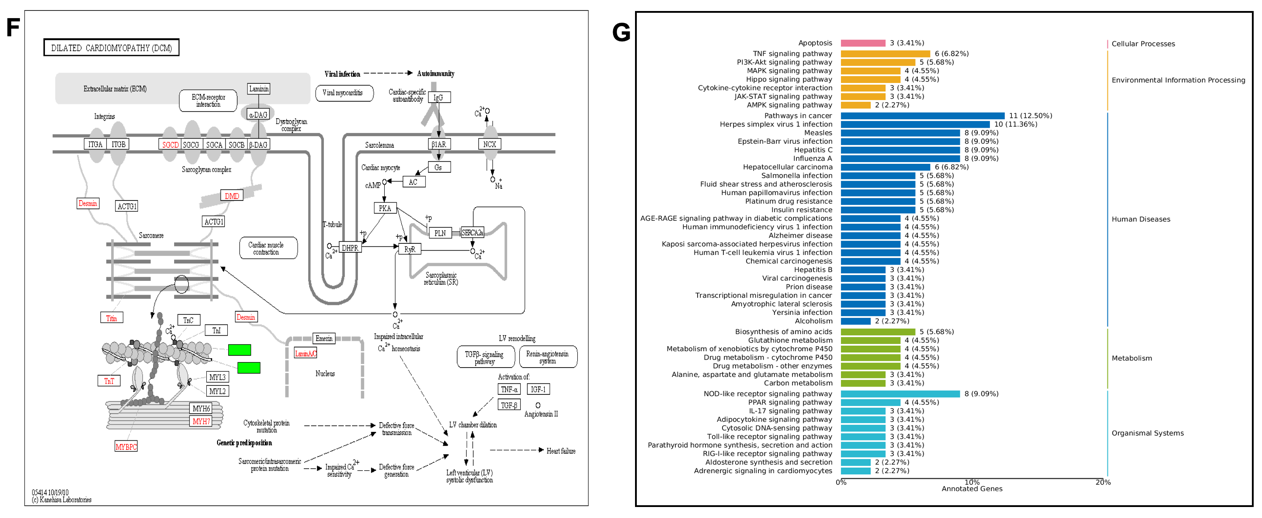


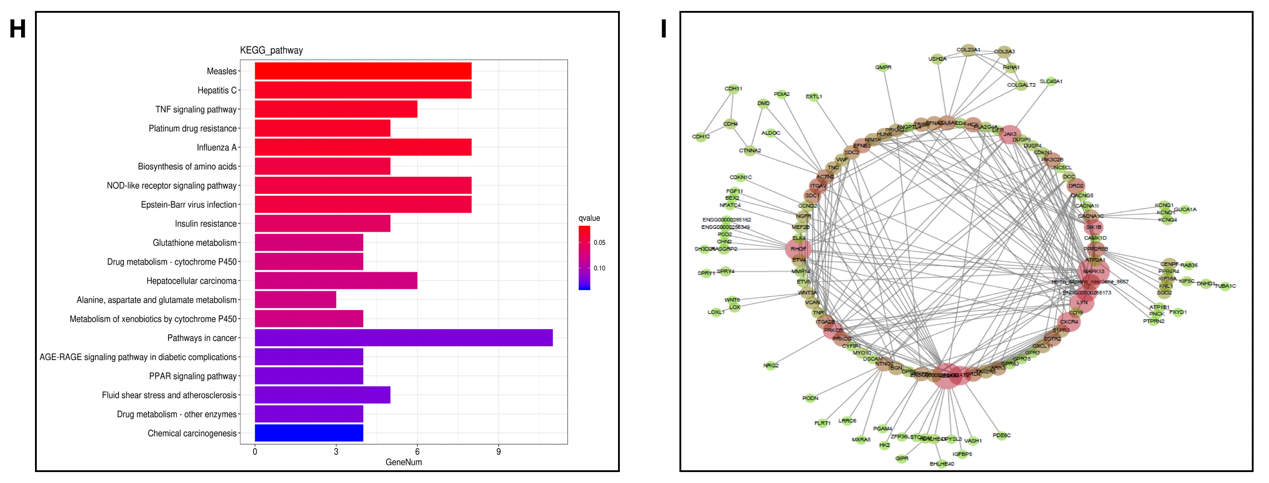


**Figure. S2:** Screening of molecules downstream of LncRNA Dleu2. **A**: Transfection efficiency and fluorescence staining of ADV-LncRNA Dleu2. **B**: Overexpression effects of LncRNA Dleu2 adenovirus. ***P<0.001. **C**: Cluster maps of differentially expressed genes (DEGs); colors represent gene expression levels in the sample. **D**: Volcano map of DEGs. **E**: Statistical map of GO-annotation classifications of DEGs. **F**: Statistical map of GO-annotation classifications of DEGs. **G**: KEGG-classification map of DEGs. **H**: KEGG-enrichment histogram of DEGs. **I**: Diagram of protein-interaction network of DEGs; the nodes are proteins and the edges are interaction relationships. ***P<0.001.


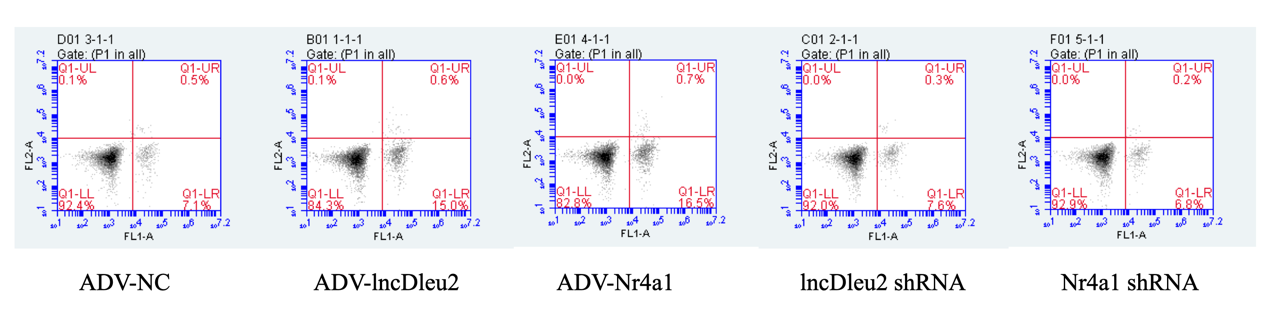


**Figure. S3:** Representative flow-cytometric image depicting cardiomyocyte apoptosis in the various groups


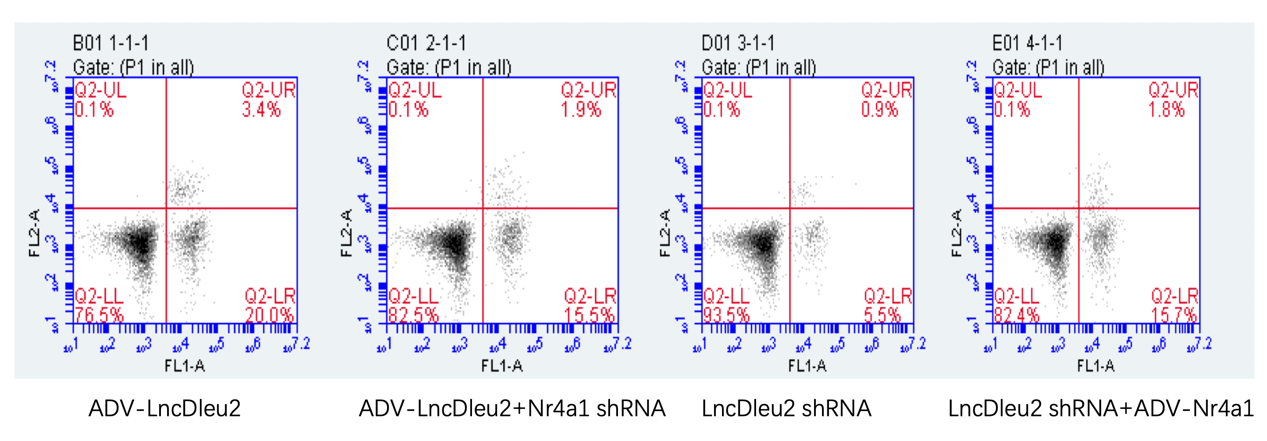


**Figure. S4:** Representative flow-cytometric image depicting cardiomyocyte apoptosis in the various groups.

**Figure S5**: The expression level of LncRNA Dleu2 and Nr4a1. **A**: The LncRNA Dleu2 expression level on day0, day 3, day 7 and day 10 after injection the adenovirus. **B**: The LncRNA Dleu2 expression level on day 0, day 3, day 7 and day 10 after injection the adenovirus. ^***^<0.001 *vs* day0.


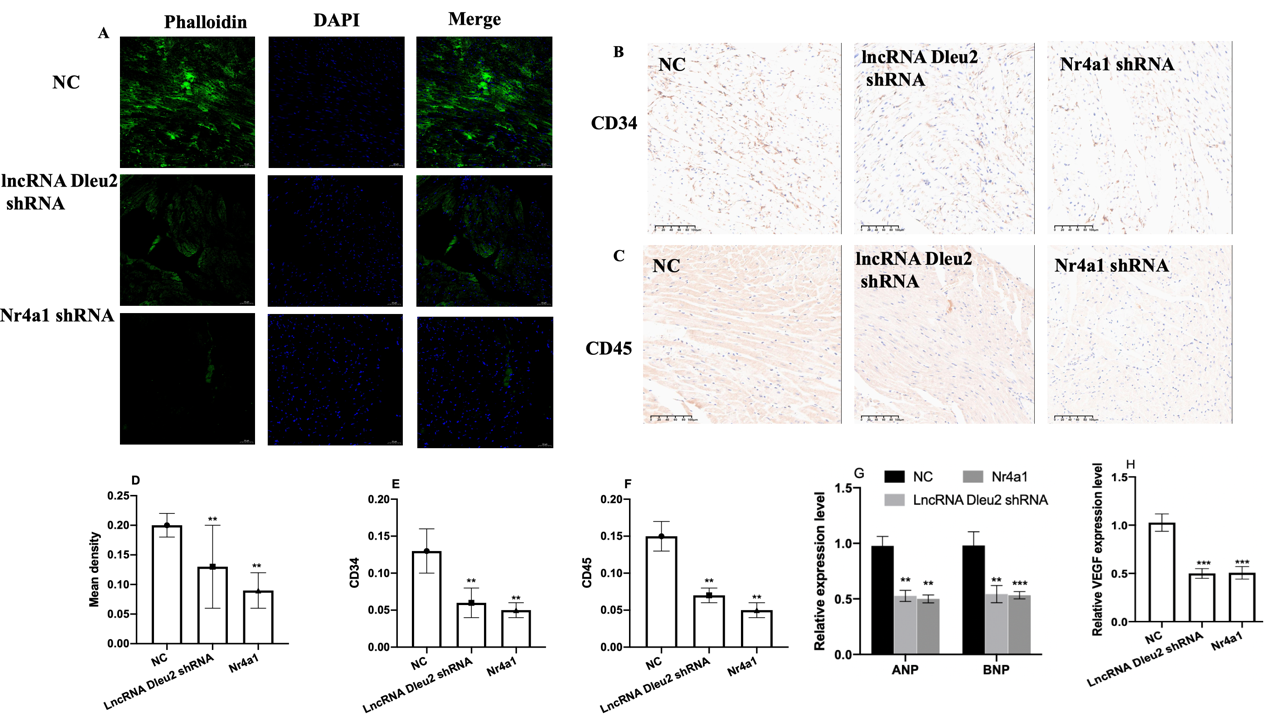


**Figure S6**: Biological function of LncRNA Dleu2 and Nr4a1 on the cardiomyocyte size, angiogenesis, inflammatory infiltration. A: Phalloidin staining showed cardiomyocyte size, the nucleus was stained with DAPI. B: Immunohistochemical staining of CD34 protein. C: Immunohistochemical staining of CD45 protein. D: The statistical graph of Phalloidin staining. E: The statistical graph of CD34. F: The statistical graph of CD45. G: The expression level of ANP and BNP. G: The expression level of VEGF. **P<0.01, ***P<0.001 vs. ADV-NC.


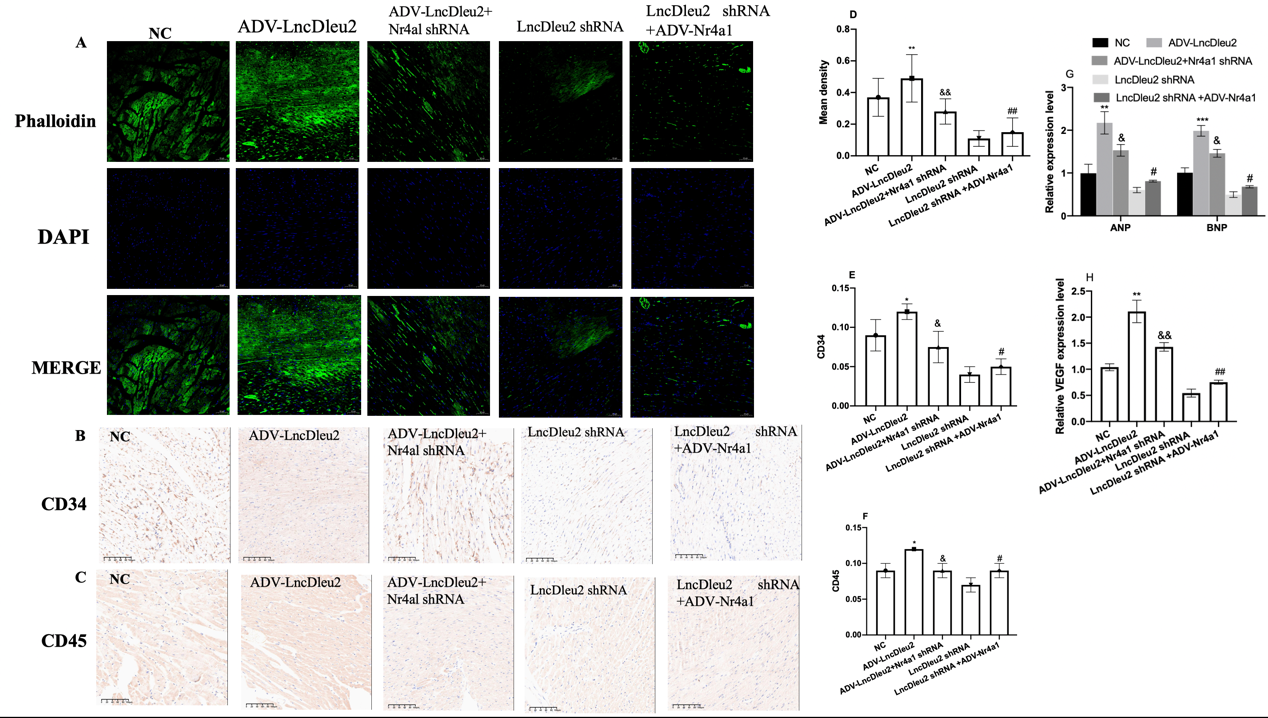


**Figure S7**: Intervention Nr4a1 expression reversed the biological function (on the cardiomyocyte size, angiogenesis, inflammatory infiltration) of LncRNA Dleu2 in vivo. A: Phalloidin staining showed cardiomyocyte size, the nucleus was stained with DAPI. B: Immunohistochemical staining of CD34 protein. C: Immunohistochemical staining of CD45 protein. D: The statistical graph of Phalloidin staining. E: The statistical graph of CD34. F: The statistical graph of CD45. G: The expression level of ANP and BNP. G: The expression level of VEGF. *P<0.05, **P<0.01vs. NC; &P<0.05, &&P<0.01vs. ADV-lncDleu2; #P<0.05, ##P<0.01vs. lncDleu2 shRNA.

**References**

1. Kirchhof P, Benussi S, Kotecha D, Ahlsson A, Atar D, Casadei B, et al (2016). 2016 ESC Guidelines for the management of atrial fibrillation developed in collaboration with EACTS. Eur Heart J 37(38):2893-2962. doi:10.1093/eurheartj/ehw210

2. Wei F, Ren W, Zhang X, Wu P, Fan J (2022). miR-425-5p is negatively associated with atrial fibrosis and promotes atrial remodeling by targeting CREB1 in atrial fibrillation. J Cardiol 79(2):202-210. doi:10.1016/j.jjcc.2021.09.012

3. Lin K, Yang N, Luo W, Qian JF, Zhu WW, Ye SJ, et al (2022). Direct cardio-protection of Dapagliflozin against obesity-related cardiomyopathy via NHE1/MAPK signaling. Acta Pharmacol Sin 43(10):2624-2635. doi:10.1038/s41401-022-00885-8

4. Tian X, Sun C, Wang X, Ma K, Chang Y, Guo Z, et al (2020). ANO1 regulates cardiac fibrosis via ATI-mediated MAPK pathway. Cell Calcium 92:102306. doi:10.1016/j.ceca.2020.102306

5. Buffolo F, Tetti M, Mulatero P, Monticone S (2022). Aldosterone as a Mediator of Cardiovascular Damage. Hypertension 79(9):1899-1911. doi:10.1161/HYPERTENSIONAHA.122.17964

6. He L, Yuan L, Yu W, Sun Y, Jiang D, Wang X, et al (2020). A Regulation Loop between YAP and NR4A1 Balances Cell Proliferation and Apoptosis. Cell Rep 33(3):108284. doi:10.1016/j.celrep.2020.108284

7. Palumbo-Zerr K, Zerr P, Distler A, Fliehr J, Mancuso R, Huang J, et al (2015). Orphan nuclear receptor NR4A1 regulates transforming growth factor-beta signaling and fibrosis. Nat Med 21(2):150-158. doi:10.1038/nm.3777

8. Liu X, Wang Y, Lu H, Li J, Yan X, Xiao M, et al (2019). Genome-wide analysis identifies NR4A1 as a key mediator of T cell dysfunction. Nature 567(7749):525-529. doi:10.1038/s41586-019-0979-8
